# Integration Viewpoint Using UHPLC-MS/MS, In Silico Analysis, Network Pharmacology, and In Vitro Analysis to Evaluate the Bio-Potential of *Muscari armeniacum* Extracts

**DOI:** 10.3390/molecules30132855

**Published:** 2025-07-04

**Authors:** Nilofar Nilofar, Gokhan Zengin, Mehmet Veysi Cetiz, Evren Yildiztugay, Zoltán Cziáky, József Jeko, Claudio Ferrante, Tina Kostka, Tuba Esatbeyoglu, Stefano Dall’Acqua

**Affiliations:** 1 Department of Biology, Science Faculty, Selcuk University, Konya 42130, Türkiye; nelofarkhattak@gmail.com; 2 Department of Pharmacy, Botanic Garden “Giardino dei Semplici”, Università degli Studi “Gabriele d’Annunzio”, Via dei Vestini 31, 66100 Chieti, Italy; claudio.ferrante@unich.it; 3 Department of Medical Biochemistry, Faculty of Medicine, Harran University, Sanliurfa 63290, Türkiye; 4 Department of Biotechnology, Science Faculty, Selcuk University, Konya 42130, Türkiye; eytugay@gmail.com; 5 Agricultural and Molecular Research and Service Institute, University of Nyíregyháza, 001 Nyíregyháza, Hungary; cziaky.zoltan@nye.hu (Z.C.); jjozsi@gmail.com (J.J.); 6 Department of Molecular Food Chemistry and Food Development, Institute of Food and One Health, Gottfried Wilhelm Leibniz University Hannover, Am Kleinen Felde 30, 30167 Hannover, Germany; kostka@chemie.uni-kl.de (T.K.); esatbeyoglu@foh.uni-hannover.de (T.E.); 7 Division of Food Chemistry and Toxicology, Department of Chemistry, RPTU Kaiserslautern-Landau, Erwin-Schrödinger-Strasse 52, 67663 Kaiserslautern, Germany; 8 Department of Pharmaceutical and Pharmacological Sciences, University of Padova, 35131 Padua, Italy

**Keywords:** *Muscari armeniacum*, muscaroside, antioxidant, NRF2, enzyme inhibition, bioactive agents, in sillico

## Abstract

The current study investigates the chemical profiling, antioxidant activities, and enzyme inhibitory and cytotoxic potential of the water and methanolic extracts of different parts (flower, leaf, and bulb) of *Muscari armeniacum*. Chemical profiling was performed using UHPLC-MS/MS. At the same time, different in vitro assays were employed to support the results for antioxidant potential, such as DPPH, ABTS, FRAP, CUPRAC, metal chelation, and PBD, along with the measurement of total phenolic and flavonoid contents. Enzyme inhibition was investigated for cholinesterase (AChE and BChE), α-amylase, α-glucosidase, and tyrosinase enzymes. Additionally, the relative expression of NRF2, HMOX1, and YGS was evaluated by qPCR. LC-MS/MS analysis indicated the presence of some significant compounds, including apigenin, muscaroside, hyacinthacine A, B, and C, and luteolin. According to the results, the highest TPC and TFC were obtained with both extracts of the leaves, followed by the water extract (flower) and methanolic extract of the bulb. In contrast, the methanolic extract from the bulb exhibited the highest antioxidant potential using DPPH, ABTS, CUPRAC, and FRAP, followed by the extracts of leaves. In contrast, the leaf extracts had the highest values for the PBD assay and maximum chelation ability compared to other tested extracts. According to the enzyme inhibition studies, the methanolic extract from the bulb appeared to be the most potent inhibitor for all the tested enzymes, with the highest values obtained for AChE (1.96 ± 0.05), BChE (2.19 ± 0.33), α-amylase (0.56 ± 0.02), α-glucosidase (2.32 ± 0.01), and tyrosinase (57.19 ± 0.87). Interestingly, the water extract from the bulb did not inhibit most of the tested enzymes. The relative expression of *NRF2* based on qPCR analysis was considerably greater in the flower methanol extract compared to the other extracts (*p* < 0.05). The relative expression of HMOX1 was stable in all the extracts, whereas YGS expression remained stable in all the treatments and had no statistical differences. The current results indicate that the components of *M. armeniacum* (leaves, flowers, and bulb) may be a useful source of natural bioactive compounds that are effective against oxidative stress-related conditions, including hyperglycemia, skin disorders, and neurodegenerative diseases. Complementary in silico approaches, including molecular docking, dynamics simulations, and transcription factor (TF) network analysis for *NFE2L2*, supported the experimental findings and suggested possible multi-target interactions for the selected compounds.

## 1. Introduction

Oxidative stress has been involved in many complications, including diabetes, cancer, cardiovascular diseases, and neurodegenerative diseases [1,2]. It results from an overproduction of reactive oxygen/nitrogen radicals, which overwhelms the body’s endogenous antioxidant defense mechanisms, preventing them from detoxifying these toxic species [3]. An antioxidant is a substance that, at low concentrations relative to an oxidizable substrate, significantly slows down or completely inhibits the oxidation of the substrate. Antioxidants directly or indirectly protect substrates from oxidation by neutralizing reactive oxygen species (ROS), preventing their formation, or enhancing the antioxidant defense system of the cell [4]. The inhibition of such key enzymes involved in the etiology of the targeted disorders has been one of the finest strategies for the management and treatment of the diseases. For instance, AChE is a central enzyme involved in the hydrolysis of acetylcholine, and the inhibition of AChE has been the most widely embraced method of treatment for Alzheimer’s disease [5]. Furthermore, tyrosinase also catalyzes enzymatic browning in fungi, vegetables, and fruits as well as mammalian melanogenesis. Tyrosinase inhibition is a powerful approach to melanogenesis control [6]. Additionally, α-glucosidase and α-amylase inhibition have been identified as an important approach for the control of blood glucose in diabetes mellitus [7].

Medicinal plants have been used as the primary source of traditional medicinal systems for disease treatment and prevention since time immemorial. Various traditional medicinal systems, like Ayurveda, Siddha, and Unani, are used for disease treatment. Some of the traditional healing systems are still used today by most rural inhabitants [8]. There has been an increasing trend in recent decades to use therapeutically active molecules from natural sources, and some of them have been found to show promising activities in the symptoms as well as in the control of the progression and complexities related to diabetes and Alzheimer’s disease. Plant sources are gaining more importance because of their nontoxic or less toxic nature and due to few or no side effects when compared to allopathic ones [9]. Many plants and herbs have been reported to have neuroprotective and anti-diabetic properties.

The genus *Muscari* Mill. is native to the Mediterranean region and comprises about 50 species. Species of this genus are commonly used in gardens as ornamental plants. *Muscari armeniacum*, commonly known as grape hyacinth, is a bulbous herbaceous perennial of the Asparagaceae family [10,11]. *M*. *armeniacum* has deep cobalt blue flowers containing delphinidin-based anthocyanins [12]. The genus name *Muscari* comes from the Latin word muscus, meaning musk, due to some species being fragrant, such as the blue-purple flowers of *M. armeniacum* itself. This plant was considered to be a grape hyacinth or Turkish hyacinth. One values this plant for its beauty and uses in parks and gardens [13]. Its bulb is a source of food, mainly in Turkey, and it has therapeutic uses, e.g., its isolated compounds have anti-viral activity [14].

This study was designed to investigate the antioxidant, enzymatic, and cytotoxic potential of *M. armeniacum* extracts using an integrated approach that combines UHPLC-MS/MS profiling, in vitro biochemical assays, qPCR-based gene expression, and in silico methods, including network pharmacology, docking, and molecular dynamics (MD) simulations. Our aim was to identify active fractions and potential compounds that target oxidative stress and cancer-associated molecular pathways.

## 2. Results and Discussion

### 2.1. Total Phenolic and Flavonoid Content

Phenolic compounds possess diverse chemical structures and play an important role in antioxidant activity by neutralizing free radicals [15]. Consequently, the total phenolic content (TPC) and total flavonoid content (TFC) of the *M*. *armeniacum* flower, leaf, and bulb samples were assessed. The water extract of flowers exhibited a higher TPC of 26 mg gallic acid equivalent (GAE)/g, followed by the leaf water extract at 23.76 mg GAE/g, but the leaf methanol extract processed a significantly higher TFC of 27.30 mg rutin equivalent (RE)/g (Table 1). The water extract of leaves presented a TFC of 20.94 mg RE/g and a TPC of 23.76 mg GAE/g. The methanol extract of the bulb had a TPC of 20 mg GAE/g, while its water extract showed a minimal TPC of 5.64 mg GAE/g. Bulb extracts showed less measurable TFC. This analysis highlights the variation in phenolic and flavonoid contents across plant parts and extraction solvents. Ozcan et al. previously compared wild onions and found that *M*. *armeniacum* possesses a higher TPC, recorded at 88.19 mg/100 g [16].

### 2.2. Chemical Composition

Methanol and aqueous extracts of leaves, flowers, and bulbs of *M. armeniacum* were analyzed using ultra-high-performance liquid chromatography (UHPLC) coupled with an Orbitrap-based high-resolution accurate-mass (HRAM) performance MS system using electrospray ionization. UHPLC-MS phytochemical profiling confirmed the presence of 53 phytoconstituents in the analyzed *M. armeniacum* leaf extracts, 51 compounds in flower extracts, and 39 components in bulb extracts. The samples showed similar chromatographic profiles, and a wide range of compounds were characterized. In all cases, the methanol extract contained a greater number of components (see Table 2). Among the identified phytochemicals, various classes of metabolites, mainly 2,5-dideoxy-2,5-imino-DL-glycero-D-manno-heptitol, hyacinthacine A, B, C, syringic acid, citric acid, *p*-coumaric acid, asperulosidic acid, ferulic acid, apigenin, luteolin, muscariflavone, dihydroxy-4-chromanone, naringenin, trihydroxy(iso)flavone muscaroside, and/or their derivatives, were characterized.

## 3. Antioxidant Effects

Recent research links oxidative stress to various diseases, emphasizing the importance of antioxidants in health maintenance and disease prevention [17,18,19]. Natural antioxidants have gained considerable interest, particularly in nutraceuticals and cosmetics [18,20,21]. Free radical-scavenging antioxidants are crucial; yet, despite extensive research, methods for assessing their effectiveness remain inconsistent. In this study, the antioxidant activity of *M*. *armeniacum* extracts from various parts was assessed using six multiple antioxidant assays, including DPPH, ABTS, CUPRAC, FRAP, metal chelating, and PBD methods (Table 3). The results varied significantly depending on the plant part and extraction solvent used, with the highest values observed in the ABTS assay of leaf water extracts and bulb methanol extracts of 60.78 mg TE/g. This bulb extract exhibited the most potent activity in CUPRAC (60.96 mg TE/g), FRAP (46.04 mg TE/g), and DPPH (39.34 mg TE/g). In contrast, the lowest antioxidant activity was recorded in the water extract of bulbs, which had minimal DPPH (5.09 mg TE/g), ABTS (8.67 mg TE/g), CUPRAC (13.83 mg TE/g), and FRAP (10.72 mg TE/g), consistent with its very low TPC (2 mg GAE/g) and TFC (0.95 mg RE/g). However, the leaf MeOH extract exhibited the highest MCA, measuring 24.35 mg EDTAE/g, followed by the bulb water extract, measuring 23.07 mg EDTAE/g. All the tested extracts showed relatively similar activity in PBD. *Muscari* seeds and bulbs have been proven to have antioxidant activity [22,23,24]. Related species, e.g., *M*. *racemosum* and *M*. *comosum,* contain common antioxidant compounds [25,26,27]. Ozcan et al. reported that *M*. *armeniacum* contains both phenolic compounds and antioxidants (2.41%) [10,16]. *M*. *armeniacum* contains anthocyanin [28,29] and has been proven to have important antioxidant activity [12]. Recent studies showed significant antioxidant activity in *M*. *armeniacum‘s* flower ethanol extract [30] and chloroform extract [31]. However, the separate parts of water and methanol extracts of *M*. *armeniacum* have not been studied in depth for their biological or antioxidant activity.

### 3.1. Enzyme Inhibitory Effects

Alzheimer’s is complex and prevalent, affecting more than 20 million people worldwide. Yet, this disease’s treatments use the cholinergic hypothesis [32]. The main approach is to use cholinesterase inhibitors targeting AChE and BChE to help improve symptoms. The AChE and BChE inhibition activity of *M*. *armeniacum* extracts is presented in Table 4. The methanol extract showed higher enzyme inhibition activity. The highest acetylcholinesterase (AChE) and butyrylcholinesterase (BChE) inhibition was observed by the methanol extract of bulbs, measuring 1.96 mg GALAE/g and 2.19 mg GALAE/g, respectively, suggesting a neuroprotective effect [33]. All three studied parts of the water extract showed lower AChE inhibition and no BChE inhibition activity, which was consistent with its generally weaker bioactivity across other assays. Recently, Tapol et al. demonstrated moderate AChE and BChE inhibition activity of *M*. *armeniacum* ethanol extracts [30].

Tyrosinase is the key enzyme that regulates melanin biosynthesis and is a primary target in the treatment of skin hyperpigmentation disorders. Tyrosinase inhibitors are commonly used to manage these conditions, which result from excessive melanin production and accumulation [34]. Table 4 represents the tyrosinase inhibition activity of *M*. *armeniacum* extracts. Tyrosinase inhibition was also the strongest in the methanolic extract, with the bulb extract exhibiting the highest 57.19 mg KAE/g, followed by the leaf extract at 44.68 mg KAE/g and the flower extract at 42.36 mg KAE/g, suggesting a dermo-protective effect [35]. Water extracts did not show detectable tyrosinase inhibition, indicating that the active compounds responsible for this effect were likely more soluble in methanol. Consistent with the previous study of Zhang et al., there was a higher anti-tyrosinase activity of *M*. *turcicum* methanol extracts, and the water extracts showed no activity.

In the case of amylase inhibition, the methanol extracts of leaves and bulbs showed the highest inhibition activity (0.57 mmol ACAE/g and 0.56 mmol ACAE/g, respectively), while the water extracts displayed significantly lower inhibition. Glucosidase inhibition showed the most striking variation. All the studied extracts exhibited lower anti-glucosidase activity, except the bulb methanol extract (2.32 mmol ACAE/g), far surpassing all other extracts. This shows that bulb methanol extracts demonstrated an anti-diabetic effect [36]. These results are consistent with the Zhang et al. study, which showed lower anti-amylase activity of *M*. *turcicum* extracts. However, their bulb extracts showed higher anti-glucosidase activity.

This preliminary report shows *M*. *armeniacum*’s anti-tyrosinase and anti-diabetic effects. Overall, the bulb methanol extract demonstrated the strongest enzyme inhibition activity across all assays, particularly for tyrosinase and glucosidase. The leaf and flower methanol extracts showed moderate inhibition in most cases, while water extracts generally exhibited the weakest enzyme inhibition activity.

### 3.2. Cell Assays

Oxidative damage and inflammation are associated with the pathogenesis of various diseases, such as Alzheimer’s [37], Parkinson’s disease [38], ischemia [39], diabetes [40], atherosclerosis [41], and cancer [42]. Unhealthy cells produce ROS as a by-product of metabolic processes and use antioxidant defenses, particularly the hyperactivation of nuclear factor erythroid 2-related factor 2 (NRF2)-dependent genes, as “masters” of adaptation [43]. This capacity to adjust and sustain oxidative, electrophilic, and inflammatory stress is triggered by NRF2. This system includes roughly 500 genes that code for cytoprotective and antioxidant proteins and is highly reliant on the network’s expression. In particular, heme oxygenase 1 (HMOX1) has potent antioxidant and anti-apoptotic properties that boost tumor cell proliferation and treatment resistance [44]. As a transcription factor, NRF2 is expressed in many organs, where it maintains cellular defense mechanisms through its antioxidant, anti-inflammatory, and cytoprotective nature.

**Figure 1:**
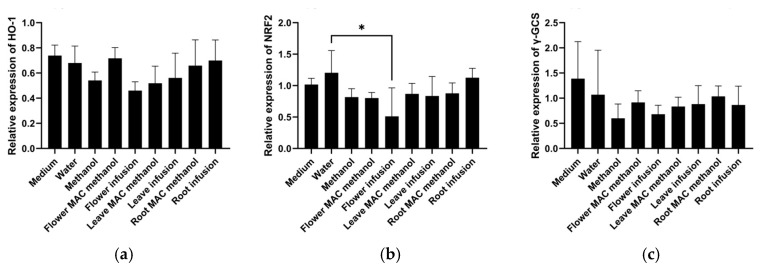
HMOX1, NRF-2, and γ-GCS gene expression was analyzed after 6 h of treatment with the different extracts of *Muscari armeniacum.* (**a**) HMOX1 expression was non-significantly reduced by the water extract of flowers; (**b**) NRF2 expression was significantly reduced by the water extract of flowers; and (**c**) γ-GCS expression was non-significantly reduced by methanol (as a solvent control) as well as the water extract of flowers. Significant differences are indicated by * (*p* < 0.05).

There are no previous studies on this topic, suggesting that the current work is the pioneer report on the expression of these important genes (HMOX1, NRF2, and γ-GCS) in HepG2 cells related to inflammation and cancer using different parts and extracts of the *M. armeniacum*. According to the results, as shown in Figure 1, the expression of HMOX1 remained relatively stable across all treatments, with minor variations. The flower water extracts significantly lowered the expression of NRF2. Other tested extract treatments exhibited intermediate levels without significant variation. The flower water extract treatments lowered the expression of γ-GCS compared to the control. The remaining extract treatments, including leaf and bulb extracts, led to moderate expression levels with no drastic differences. Overall, the water extract from the flower resulted in the lowest expression of NRF2, HMOX1, and γ-GCS, suggesting that the reduced expression of NRF2, as a transcription factor for many genes, is responsible for the lower expression of HMOX1 and γ-GCS.

A previous study found that *M*. *comosum* L. bulb extracts influence oxidative stress in HepG2 cells by altering the regulation of specific genes associated with redox and inflammation, such as NRF2 and SOD-2 [45]. Like antioxidant activity, immune modulation can also be considered a prospective property of Muscari, as it can help fight various infectious and important non-infectious diseases, including cancer [46]. However, only a few studies have demonstrated the antioxidant activity of *Muscari* species. Nevertheless, the scientific literature lacks comprehensive research on the antioxidant effects of *Muscari armeniacum* at the cellular level.

### 3.3. Transcription Factor Analysis

**Figure 2:**
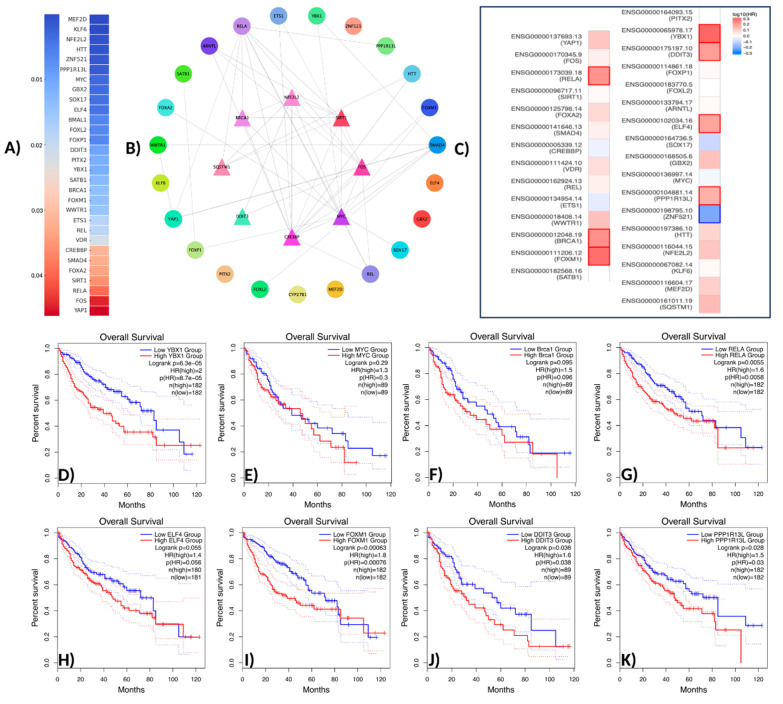
Network pharmacology workflow. (**A**) Gene analysis associated with NFE2L2 using IPA (*p*-value > 0.05). (**B**) The protein–protein interaction network of potential TFs displays the initial nodes located in the innermost circle. (**C**) Survival analysis of TFs. (**D**) Overall survival analysis of YBX1. (**E**) Overall survival analysis of MYC (c-Myc). (**F**) Overall survival analysis of Brac1. (**G**) Overall survival analysis of RELA (NF-κB p65). (**H**) Overall survival analysis of ELF4. (**I**) Overall survival analysis of FOXM1. (**J**) Overall survival analysis of DDIT3. (**K**) Overall survival analysis of PPP1R13L.

In this study, a comprehensive analysis was conducted on 31 transcription factors (TFs) associated with the *NFE2L2* gene. The transcription factors identified through the use of Ingenuity Pathway Analysis (IPA) were validated in the context of the protein–protein interaction (PPI) network using the STRING database. The validation process was then visualized using Cytoscape v3.10.3 software. The results of the biological enrichment analysis performed with ShinyGO v0.77 (http://bioinformatics.sdstate.edu/go77, access date: 16 May 2025) revealed the roles of the identified TFs in pathways within the context of LIHC. Survival analyses were evaluated using the Overall Survival method on the GEPIA2 platform, and statistically significant transcription factors were identified. The analysis identified TFs with direct interaction with *NFE2L2* and a *p*-value less than 0.05. In this context, the genes *SQSTM1*, *MEF2D*, *KLF6*, *NFE2L2*, *HTT*, *ZNF521*, *PPP1R13L*, *MYC*, *GBX2*, *SOX17*, *ELF4*, *BMAL1*, *FOXL2*, *FOXP1*, *DDIT3*, and *YBX* were found to be statistically significantly associated with NFE2L2. *PITX2*, *SATB1*, *FOXM1*, *BRCA1*, *WWTR1*, *ETS1*, *REL*, *VDR*, *CREBBP*, *SMAD4*, *FOXA2*, *SIRT1*, *RELA*, *FOS*, and *Yap1* were found to be statistically significantly associated with *NFE2L2* (Figure 2A).

**Figure 3:**
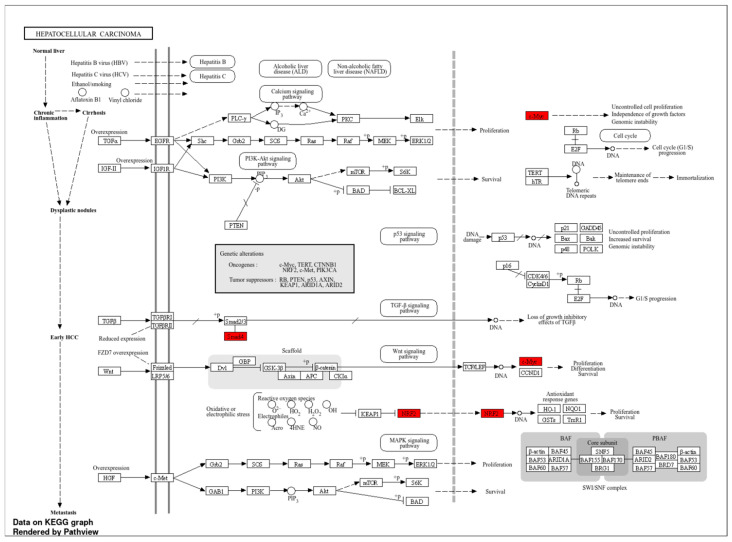
Pathways of LIHC, highlighting key genes and their interrelationships.

TFs were positioned at the core of the network, while those exhibiting indirect interactions were designated as peripheral nodes. TF–*NFE2L2* interactions were identified as direct for *FOXM1*, *RELA*, *YBX1*, *SIRT1*, *BRCA1*, *CREBBP*, *FOS,* and *SQSTM1*. The central position of these TFs in the network suggests that they play a critical role in regulating *NFE2L2*’s biological effects (Figure 2B). Furthermore, survival map analysis conducted with GEPIA2 revealed that *PPP1R13L, ELF4, DDIT3, YBX1, FOXM1, BRCA1,* and *RELA* were found to be prominent (Figure 2C). In survival analyses, *YBX1* exhibited the highest hazard ratio (HR = 2) and was identified as the most statistically significant transcription factor (*p* = 0.00005), suggesting its critical role in the proliferation and metastasis of cancer cells. Similarly, *FOXM1* (HR = 1.8, *p* = 0.001), a cell cycle regulator, plays a critical role in DNA repair and tumor growth. *RELA* (NF-κB p65) (HR = 1.6, *p* = 0.0055) has been linked to inflammatory processes and has been shown to contribute to immune suppression and chemotherapy resistance in the tumor microenvironment through the activation of NF-κB signaling pathways. *DDIT3* (hazard ratio [HR] = 1.6, *p* = 0.036) has been associated with the endoplasmic reticulum (ER) stress response, while *PPP1R13L* (HR = 1.5, *p* = 0.028) has been identified as a critical component of apoptosis mechanisms due to its interaction with p53. *BRCA1* (HR = 1.5, *p* = 0.095) may be implicated in LIHC development through its function in DNA damage response mechanisms. Furthermore, the analysis identified *MYC* (HR = 1.3, *p* = 0.29) and *ELF4* (HR = 1.4, *p* = 0.055) as transcription factors that were found to be at the significance threshold for survival and were considered potential risk factors in cancer biology (Figure 2D–K). These findings suggest that TFs associated with *NFE2L2* should be considered therapeutic targets. Survival analyses have particularly shown that *YBX1, FOXM1*, and *RELA* are associated with poor prognosis in LIHC (Figure 2D,H,K). In addition to the TFs identified in pathway analysis, other TFs that are significant in survival are also considered important regulators in cancer progression (Figure 3).

In summary, the TFs identified in this study were determined to be key molecules regulating cancer progression through *NFE2L2*. Specifically, TFs such as *YBX1*, *FOXM1*, and *RELA*, which are associated with poor prognosis, can be considered targetable biomarkers. The pharmacological inhibition of these TFs and functional validation in laboratory settings will facilitate the development of next-generation therapeutic approaches for hepatocellular carcinoma.

### 3.4. Molecular Docking

**Figure 4:**
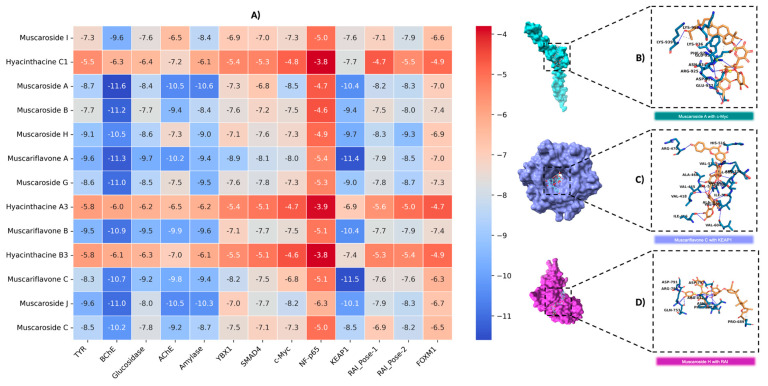
An analysis of binding interactions between example compounds and proteins. (**A**) Graphical representation of docking scores for all targets. (**B**) Molecular interaction analysis of muscaroside A with c-Myc. (**C**) Molecular interaction analysis of muscariflavone C with KEAP1. (**D**) Molecular interaction analysis of muscaroside H with RAI.

In this study, the binding affinities of muscaroside and muscariflavone derivatives were comprehensively evaluated against a diverse set of targets, including AChE, BChE, amylase, glucosidase, tyrosinase, KEAP1, c-Myc, YBX1, SMAD4, NF-p65 (RELA), FOXM1, and RAI. The efficacy of these molecules was assessed based on binding energy (kcal/mol), the number of hydrogen bonds, and RMSD values. Compounds with an RMSD of 2 Å or lower were considered to adopt stable conformations suitable for target proteins. Among the compounds that were analyzed, muscariflavone A exhibited the lowest RMSD value of 0.07 Å when bound to c-Myc, whereas muscaroside G showed the highest RMSD value of 15.8 Å with RAI-Pose-2. Muscaroside J exhibited the lowest number of hydrogen bonds, with a total of five interactions observed in the complex with amylase. Conversely, muscariflavone A demonstrated the highest number of hydrogen bonds, forming twenty-two interactions with tyrosinase. To further refine the selection, a binding energy threshold of −8 kcal/mol or lower was applied as the cutoff for subsequent analyses (Table S2 and Figure 4A).

The following compounds were analyzed for AChE inhibition: muscaroside A, muscariflavone A, muscariflavone B, muscariflavone C, muscaroside J, and muscaroside C. Of these, muscaroside A exhibited the highest binding affinity, indicating a stable interaction with AChE. Furthermore, muscariflavone B and C exhibited a greater propensity for hydrogen bond formation, while muscaroside J and muscariflavone A, despite their reduced hydrogen bond formation, demonstrated robust binding capabilities. This observation suggests the potential for inhibitory effects to be exerted via shared hotspot residues. In this study, the compounds subjected to evaluation included muscaroside A, B, H, J, C, and muscariflavone A, B, and C. The results indicate that muscaroside A exhibited the strongest affinity, while muscaroside B and muscariflavone A demonstrated the capacity to form the most hydrogen bonds. Despite its reduced number of H-bonds, muscaroside J exhibited robust and consistent binding, engaging with critical active site residues. The findings of this study suggest that muscaroside and muscariflavone derivatives may act as potent cholinesterase inhibitors via similar binding mechanisms.

The following compounds were analyzed for amylase inhibition: muscaroside I, muscaroside H, muscaroside J, muscaroside A, muscaroside B, muscaroside C, and muscaroside J. Among these, muscaroside J demonstrated the strongest and most stable inhibition, exhibiting the highest binding affinity and the lowest RMSD value. Muscaroside I and muscari-flavone B exhibited a high frequency of hydrogen bond formation; yet, these compounds possessed comparatively lower binding energies. In contrast, muscariflavone A and C demonstrated moderate inhibitory activity. Key hotspot residues for ligand binding were identified as ILE A: 148, TYR A: 151, THR A: 163, and HIS A: 305. The glucosidase inhibition potential of muscaroside A, muscaroside H, and muscariflavone A, B, and C was subsequently analyzed. Muscariflavone A was identified as the most potent inhibitor based on its high binding energy and low root mean square deviation (RMSD) value. In contrast, muscariflavone B and C exhibited the highest number of hydrogen bonds. The binding affinities of muscaroside A and H were found to be lower, suggesting that these compounds engage in less stable interactions.

Muscariflavone C showed the highest binding affinity and number of hydrogen bonds for KEAP1 inhibition (Figure 4C). For c-Myc, muscaroside A (Figure 4B) had the strongest binding affinity, while muscariflavone A exhibited the most stable binding pose (RMSD: 0.07 Å). In the YBX1 target, muscariflavone A had a stronger binding affinity, while muscariflavone C formed more hydrogen bonds. For SMAD4, muscariflavone A had the highest affinity, and muscaroside G showed strong hydrogen bonding. In RAI inhibition, muscaroside H (Figure 4D) was the most effective in both poses, showing high binding affinities; for Pose-1, it showed key interactions with ASP A:633, ASN A:666, and HIS A:692. These results highlight the multi-target inhibitory potential of muscaroside and muscariflavone derivatives.

These findings demonstrate that muscaroside and muscariflavone derivatives exhibit significant binding affinities and stable interactions with a broad range of therapeutic targets, including but not limited to AChE, BChE, KEAP1, c-Myc, amylase, glucosidase, tyrosinase, RAI, and others, involved in neurodegenerative, metabolic, oxidative, and oncogenic pathways. The substantial concordance between the docking results and the in vitro bioactivity data for multiple targets, particularly cholinesterases and glucosidases, underscores the translational significance of these compounds. The capacity of these phytochemicals to modulate a variety of protein targets indicates a promising avenue for multi-target drug development. Collectively, our integrated computational and experimental data support further preclinical evaluation of muscaroside and muscariflavone derivatives, including comprehensive pharmacokinetic and in vivo efficacy studies, to better define their therapeutic potential.

### 3.5. Molecular Dynamics Simulation

**Figure 5:**
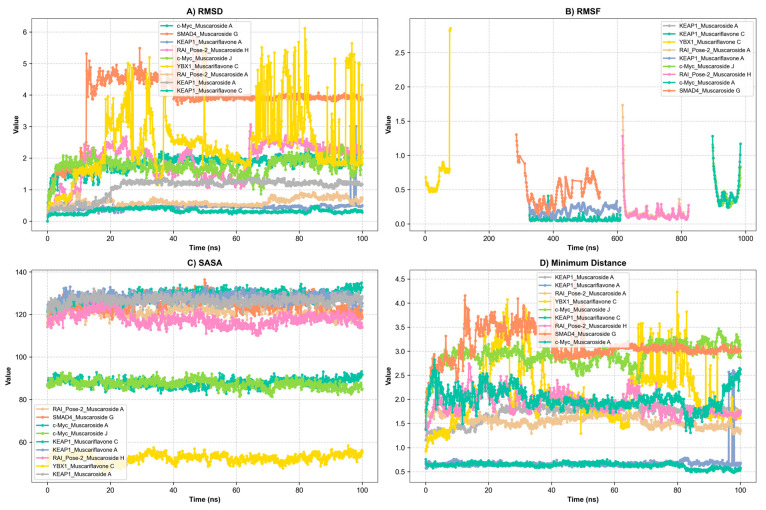
Presentation of molecular dynamics simulations in graphical form: (**A**) RMSD of KEAP1_Muscaroside A, KEAP1_Muscariflavone A, KEAP1_Muscariflavone C, RAI_Pose-2_Muscaroside, c-Myc_Muscaroside J, KEAP1_Muscariflavone C, RAI_Pose-2_Muscaroside A, SMAD4_Pose-2_Muscaroside G, YBX1_Muscariflavone C, and c-Myc_Muscaroside A complexes. (**B**) RMSF of KEAP1_Muscaroside A, KEAP1_Muscariflavone A, KEAP1_Muscariflavone C, RAI_Pose-2_Muscaroside, c-Myc_Muscaroside J, KEAP1_Muscariflavone C, RAI_Pose-2_Muscaroside A, SMAD4_Pose-2_Muscaroside G, YBX1_Muscariflavone C, and c-Myc_Muscaroside A complexes. (**C**) Solvent accessibility of KEAP1_Muscaroside A, KEAP1_Muscariflavone A, KEAP1_Muscariflavone C, RAI_Pose-2_Muscaroside, c-Myc_Muscaroside J, KEAP1_Muscariflavone C, RAI_Pose-2_Muscaroside A, SMAD4_Pose-2_Muscaroside G, YBX1_Muscariflavone C, and c-Myc_Muscaroside A complexes. (**D**) Minimum distance of KEAP1_Muscaroside A, KEAP1_Muscariflavone A, KEAP1_Muscariflavone C, RAI_Pose-2_Muscaroside, c-Myc_Muscaroside J, KEAP1_Muscariflavone C, RAI_Pose-2_Muscaroside A, SMAD4_Pose-2_Muscaroside G, YBX1_Muscariflavone C, and c-Myc_Muscaroside A complexes.

**Figure 6:**
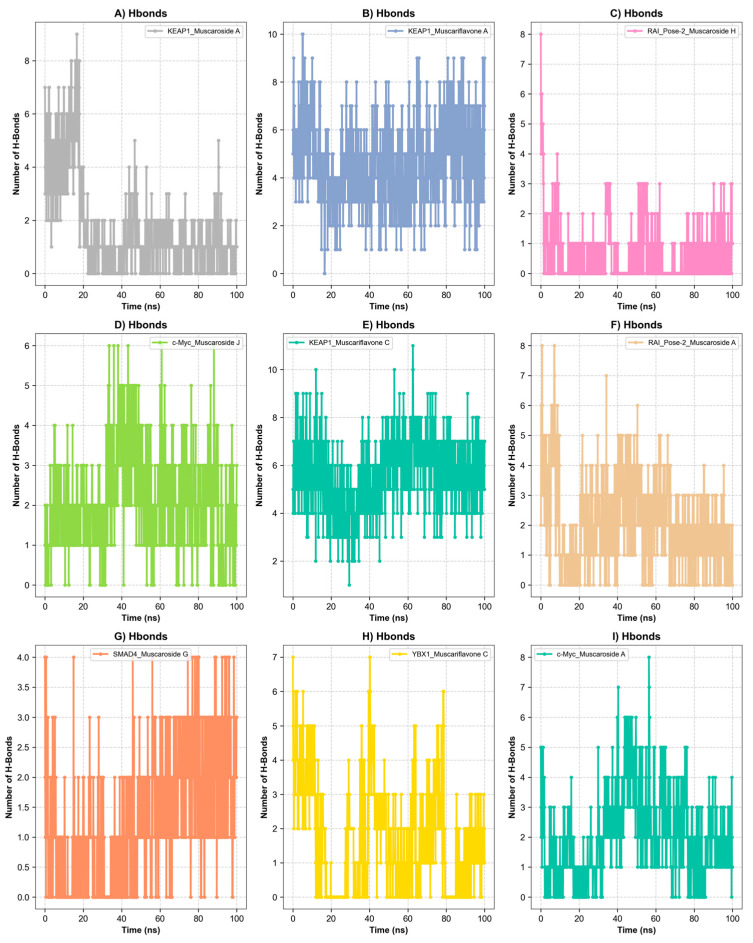
Hydrogen bond analysis over a 100 ns simulation. (**A**) Hydrogen bond of KEAP1_Muscariflavone A complex. (**B**) Hydrogen bond of KEAP1_Muscariflavone C complex. (**C**) Hydrogen bond of RAI_Pose-2_Muscaroside complex. (**D**) Hydrogen bond of c-Myc_Muscaroside J. (**E**) Hydrogen bond of KEAP1_Muscariflavone C complex. (**F**) Hydrogen bond of RAI_Pose-2_Muscaroside A complex. (**G**) Hydrogen bond of SMAD4_Pose-2_Muscaroside G complex. (**H**) Hydrogen bond of YBX1_Muscariflavone C complex. (**I**) Hydrogen bond of c-Myc_Muscaroside A complex.

**Figure 7:**
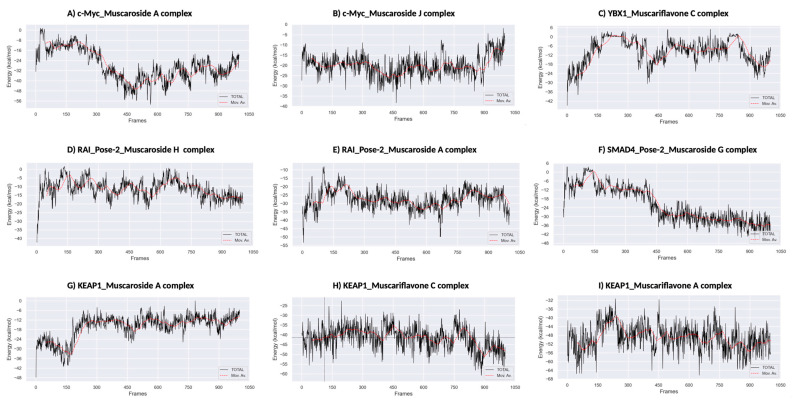
MM/PBSA binding free energy calculations. (**A**) KEAP1_Muscariflavone A complex. (**B**) KEAP1_Muscariflavone C complex. (**C**) RAI_Pose-2_Muscaroside complex. (**D**) c-Myc_Muscaroside J. (**E**) KEAP1_Muscariflavone C complex. (**F**) RAI_Pose-2_Muscaroside A complex. (**G**) SMAD4_Pose-2_Muscaroside G complex. (**H**) YBX1_Muscariflavone C complex. (**I**) c-Myc_Muscaroside A complex.

A total of ten ligand–protein complexes, encompassing KEAP1, RAI, c-Myc, SMAD4, and YBX1 targets, were subjected to 100 ns MD simulations to evaluate stability and dynamic binding behavior (Figure 5, Figure 6 and Figure 7). Across all systems, ligand retention, RMSD, RMSF, hydrogen bonding, and SASA were monitored and are summarized in Figure 5A–D and Figure 6. MM/GBSA binding free energy values (Figure 7) provided a quantitative thermodynamic assessment.

Among all complexes, KEAP1_Muscariflavone A, KEAP1_Muscariflavone C, and RAI_Muscaroside A (Pose-2) exhibited the highest stability, as evidenced by a consistently low root mean square deviation (RMSD; 0.32–0.60 Å; Figure 5A), limited residue fluctuations (RMSF; Figure 5B), persistent ligand–protein distances (Figure 5D), and stable hydrogen bond trends (Figure 6). The maintenance of moderate SASA (121–128 nm^2^; Figure 5C) and highly negative MM/PBSA ΔG values (Figure 7H,I,E) was also confirmed, thereby validating the strength and thermodynamically favorable binding profiles of the systems. Conversely, SMAD4_Muscaroside G (Pose-2) exhibited augmented RMSD (~3.81 Å) and RMSF (~0.46 Å) (Figure 5A,B), denoting heightened flexibility, yet maintained negative binding energy (Figure 7F) and augmented hydrogen bonding after 50 ns (Figure 6G).

c-Myc_Muscaroside A displayed intermediate stability, with RMSD rising from 1.03 Å to 1.88 Å (Figure 5A), modest residue flexibility (Figure 5B), and moderate binding energy (~−24 kcal/mol; Figure 7A). c-Myc_Muscaroside J exhibited weaker stability, with a higher RMSD (1.74 Å), lower hydrogen bond persistence (Figure 6H), and borderline ΔG (−10 kcal/mol; Figure 7B).

A more pronounced degree of instability was observed for YBX1_Muscariflavone C (RMSD up to 2.7 Å, RMSF peaks > 2.8 Å; Figure 5A,B), a decline in hydrogen bonds (Figure 6H), and positive binding energy (Figure 7C), suggesting transient interactions. RAI_Muscaroside H (Pose-2) exhibited an analogous pattern of early RMSD increase (2.01 Å), moderate SASA (118 nm^2^; Figure 5C), and less favorable binding energy (−15 kcal/mol; Figure 7D). KEAP1_Muscaroside A exhibited a mixture of stability characteristics, characterized by an increase in RMSD (1.07 Å), a reduction in H-bond persistence (Figure 6A), and a positive ΔG (Figure 7G).

The MD and MM/GBSA analyses (Figure 5, Figure 6 and Figure 7) underscore the dynamic stability and strong binding of selected muscaroside and muscariflavone derivatives—especially in complexes such as KEAP1_Muscariflavone A/C and RAI_Muscaroside A—across multiple evaluation parameters. These results align with the docking predictions and in vitro bioactivity data, highlighting the potential of these phytochemicals as multi-target modulators relevant to neurodegeneration, metabolic, and oncogenic diseases. Conversely, complexes displaying high flexibility or positive binding energy (YBX1_Muscariflavone C) may have limited inhibitory potential. Overall, the integrated figure-supported analysis identifies robust candidates and justifies further preclinical investigation.

## 4. Materials and Methods

### 4.1. Plant Collection

In 2020, botanical specimens were collected from the city forest of Konya (Beykonagi location, Ilgın, 1410 m), Türkiye. Dr. Evren Yildiztugay conducted the taxonomic identification, and a voucher specimen was preserved in the herbarium of Selcuk University (Voucher number: EY-2977). The plant parts (flowers, leaves, and bulbs) were carefully separated and dried in the shade at ambient temperature, powdered, and thereafter stored away from light.

### 4.2. Plant Extract Preparation

The extraction procedure included two solvents: methanol and water. Each 10 g sample was macerated with 200 mL of methanol for 24 h at room temperature. The aqueous extracts were prepared by infusing 10 g of plant material in boiling water for 15 min. Organic solvents were removed via evaporation under low pressure, and the aqueous extract was subjected to freeze-drying.

### 4.3. Spectrophotometric Assay for Total Phenolic and Flavonoid Contents

Total phenolics and flavonoids were quantified in our previous paper [47]. Gallic acid and rutin served as reference standards in the experiments, with results reported as gallic acid equivalent (GAE) and rutin equivalent (RE).

### 4.4. UHPLC-MS/MS Metabolomic Analysis

The phytochemical analysis of leaf, flower, and bulb extracts of *Muscari armeniacum* was performed using ultra-high-performance liquid chromatography (UHPLC) coupled with an electrospray ionization source and an Orbitrap-based high-resolution accurate-mass (HRAM) performance MS system (Thermo Q-Exactive, Thermo Scientific, Waltham, MA, USA). A previously developed and successfully applied gradient UHPLC separation was carried out on a Thermo Accucore analytical C18 column (100 mm × 2.1 mm i.d., 2.6 μm) using a Dionex 3000RS UltiMate system (Thermo Scientific). All details are given in the Supplemental Materials [48].

### 4.5. Assays for In Vitro Antioxidant Capacity

As previously described [49], various antioxidant tests were performed. DPPH, ABTS radical scavenging, CUPRAC, and FRAP results are shown as milligrams of Trolox equivalent (TE) per gram. The phosphomolybdenum (PBD) test measured antioxidant potential in millimoles of TE per gram of extract, and metal chelating activity (MCA) was measured in mg EDTAE.

### 4.6. Inhibitory Effects Against Some Key Enzymes

Enzyme inhibition studies were performed on samples according to the method in [49]. Acarbose equivalent (ACAE) per gram of extract inhibited amylase and glucosidase, while milligrams of galanthamine equivalent (GALAE) inhibited acetylcholinesterase (AChE) and butyrylcholinesterase. To evaluate tyrosinase inhibition per gram of extract, milligrams of kojic acid equivalent (KAE) were used.

### 4.7. Cell Cultivation

Human hepatoblastoma cells (HepG2) (DSMZ, Braunschweig, Germany) were maintained in T75 flasks (Sarstedt, Germany) with 15 mL DMEM (Pan-Biotech, Germany) supplemented with 10% fetal bovine serum and 5% antibiotics (penicillin 10,000 U/mL; streptomycin 10 mg/mL). Cultures were incubated at 37 °C in 5% CO_2_ (Heracell™ VIOS160i, ThermoFisher Scientific, Darmstadt, Germany). The medium was refreshed three times per week. For passaging, cells were detached once weekly using 2 mL trypsin-EDTA, incubated for 8 min at 37 °C, and then centrifuged at 5000× *g* for 5 min. The supernatant was discarded, and the pellet was resuspended in 2 mL DMEM. A 200 µL aliquot was seeded into a new T75 flask.

### 4.8. Cytotoxicity Assay

A resazurin cytotoxicity assay verified that test materials were not cytotoxic to HepG2 cells. HepG2 cells were planted at 5000 per well in 60 wells in a Sarstedt 96-well plate. Cells were cultivated for 24 h before treatment. After 24 h, cells were treated with 50 µg L^−1^ and 75 µg L^−1^ extracts for 24 h. The negative control was 1% Triton X. Resveratrol was employed as a positive control at 25 and 50 µM doses. Then, 24 h after incubation, treatment solutions were removed, and 100 µL of 10% resazurin solution was added to wells. The CO2 incubator incubated the plate at 37 °C for 4 h. A multiplate reader (Infinite M200, Tecan, Männedorf, Switzerland) detected the fluorescence signal (excitation wavelength of 560 nm and emission wavelength of 690 nm) after incubation.

### 4.9. Cell Culture Experiments for Subsequent qPCR Analysis

HepG2 cells were seeded at 1 × 10^6^ per well in 6-well plates (Sarstedt, Nümbrecht, Germany) for gene expression analysis. Cells were cultivated for 24 h before treatment. Following this, cells were treated with 100 µg/mL plant extracts. After a 6-h treatment, cells were washed with 500 µL of PBS (ChemSolute, Renningen, Germany). Cells were detached from wells using 250 µL trypsin-EDTA (Pan-Biotech, Aidenbach, Germany) and incubated at 37 °C in a ThermoFisher Scientific CO_2_ incubator for 6 min. The cell suspension was then placed in a 2 mL tube and centrifuged for 5 min at 1000× *g*. The supernatant was collected, and pellets were stored at −80 °C until RNA extraction.

### 4.10. RNA Extraction

Total RNA was extracted using phenol/chloroform. Cell pellets were homogenized in 1 mL of a mixture containing 48% water-saturated phenol, 33% deionized water, 5% glycerol, 3.5% NaAc buffer (pH = 5.0), 0.4 M ammonium thiocyanate, and 0.8 M guanidium thiocyanate to extract RNA (Applichem, Darmstadt, Germany). Cell pellets were shaken at room temperature for 30 min (CellMedia, Zeitz, Germany) to homogenize them. Next, 200 µL chloroform (Sigma-Aldrich, Taufkirchen, Germany) was added to the mixture, inverted manually for 15 s, and incubated at room temperature for 3 min. Tubes were centrifuged for 15 min at 12,000× *g* at 4 °C. Post-centrifugation, 400 µL of the upper phase was mixed with 500 µL of isopropanol in a fresh 1.5 mL tube (Walter-CMP, Kiel, Germany). The mixture was inverted 10–15 times, incubated at ambient temperature for 10 min, and centrifuged at 12,000× *g* for 10 min at 4 °C. The supernatant was removed, and the pellet was washed with 1 mL 70% ethanol (Walter, Kiel, Germany), shaken for 10 min on a shaker, then centrifuged at 7500× *g* at 4 °C for 5 min. Following pipette removal of ethanol, the pellet was left to dry. The RNA was resuspended by resolving the pellet in 25 µL of nuclease-free water (Life Technologies, Darmstadt, Germany). Samples of RNA were kept at −20 °C until analysis.

### 4.11. Synthesis of cDNA

RNA was diluted 1:10 in nuclease-free water. Afterward, randomly selected samples were diluted 1:4 and 1:16 (*v*/*v*) to confirm cDNA synthesis linearity. To remove genomic DNA, 2 µL diluted RNA was mixed with 1.2 µL DNase, 1.2 µL DNase buffer, and 7.6 µL nuclease-free water in a 96-well plate (Sarstedt, Nümbrecht, Germany). A thermal shaker was used to incubate the plate at 37 °C for 30 min after closing it with an adhesive cover (Sarstedt). Incubating the plate at 70 °C for 15 min degraded DNase. After cooling on ice, the plate was briefly centrifuged, and 1 µL of 50 pmol µL^−1^ random nonamer primer was applied. Incubating the plate at 70 °C for 5 min resolved secondary nucleic acid structures. The primer was annealed by chilling the plate on ice immediately. Then, 0.5 µL reverse transcriptase (Promega, Walldorf, Germany), 4 µL buffer, 2 µL of 10 mM dNTPs (Thermo Scientific), and 0.5 µL nuclease-free water were added after brief centrifugation. Reverse transcriptase was replaced with nuclease-free water in random samples to remove genomic DNA. After brief centrifugation, reverse transcription was performed at 37 °C for 1 h, and enzymes were inactivated at 70 °C for 15 min. Samples were kept at −20 °C until analysis.

### 4.12. Quantitative Polymerase Chain Reaction (qPCR)

To quantify RNA, 2 µL of each sample was pooled and diluted using a 1:4 dilution factor to create a standard curve using transcription data. For qPCR analysis, 2 µL cDNA, 2.2 µL nuclease-free water, 5 µL SYBR Green Mix (Applied Biosystems, Darmstadt, Germany), and 0.4 µL forward and reverse primer (Eurofins, Hamburg, Germany) were added to a white 96-well plate (Sarstedt). A thermocycler (QuantStudio3 Real-Time PCR System; ThermoFisher Scientific, Darmstadt, Germany) was used for qPCR analysis. Each of the 40 PCR cycles involved the following steps: 95 °C for 10 min and 60 °C for 60 s. The dissociation curve was recorded after 40 cycles at 95 °C for 15 s, 60 °C for 60 s, and 95 °C at 0.15 °C s^−^^1^.

### 4.13. TF Analysis

Transcriptional factors (TF) associated with the *NFE2L2* gene were identified using QIAGEN Ingenuity Pathway Analysis (IPA) and prioritized based on a significance threshold of *p*-value ≤ 0.05. The identified transcription factors were further validated and visualized using Cytoscape v3.10.3 with the STRING database plugin (https://string-db.org/ accessed on 18 May 2025). Subsequently, these validated transcription factors were subjected to biological enrichment analysis using ShinyGO v0.77 (http://bioinformatics.sdstate.edu/go77/ accessed on 18 May 2025). The analysis focused on hepatocellular carcinoma (LIHC). Subsequently, the complete set of transcription factors was analyzed for overall survival and a survival map using the GEPIA2 platform (http://gepia2.cancer-pku.cn/#index, accessed on 18 May 2025), applying a significance threshold of *p*-value ≤ 0.05 with a 50% cutoff for both high- and low-expression groups. The survival analysis results were assessed to validate the clinical relevance of the transcription factors. Pathway analysis was conducted using the *pathview* v1.46.0 package in R version 4.3.3 software (https://bioconductor.org/packages/release/bioc/html/pathview.html, accessed on 18 May 2025).

### 4.14. Protein and Ligand Preparation

Molecular docking analysis was conducted to evaluate the interactions of the major phytochemicals present in *M. armeniacum* extracts. The 3D structures of the selected target proteins, identified through the Pharos platform (https://pharos.nih.gov/, accessed on 18 May 2025), were retrieved from the Protein Data Bank (PDB) (https://www.rcsb.org/, accessed on 18 May 2025) using their corresponding PDB IDs: AChE (PDB ID: 2Y2V), BChE (PDB ID: 3DJY) [50], amylase (PDB ID: 2QV4), glucosidase (PDB ID: 3W37) [51], and tyrosinase (PDB ID: 5M8O). Furthermore, molecular docking was performed for *NFE2L2* gene related-proteins, including FOXM1 (PDB ID: 3G73), RAI (PDB ID: 4V53), KEAP1 (PDB ID:6T7V), c-MYC (PDB ID: 1NKP) [52], SMAD4 (PDB ID: 1DD1), and YBX1 (PDB ID: 6KTC). All 3D structures of ligands were downloaded from the PubChem database (https://pubchem.ncbi.nlm.nih.gov/, accessed on 18 May 2025) and optimized using Avogadro V1.2.0. The three-dimensional (3D) ligand structures were initially obtained from the PubChem database (https://pubchem.ncbi.nlm.nih.gov/, accessed on 18 May 2025). For ligands that were not available in PubChem, the IUPAC names were retrieved from both the SpectraBase database (https://spectrabase.com/, accessed on 18 May 2025) and literature. SMILES representations were then generated via Chemaxon (https://www.rcsb.org/chemical-sketch, accessed on 18 May 2025). The resulting SMILES strings were then converted to PDB format using Novopro (https://www.novoprolabs.com/tools/smiles2pdb, accessed on 18 May 2025). Additionally, for ligands absent in both PubChem and SpectraBase, the OPSIN (Indigo Toolkit) service (https://opsin.ch.cam.ac.uk/, accessed on 18 May 2025) was employed, and the structures were subsequently optimized using Avogadro V1.2.0. This workflow ensures that each ligand is provided in a consistent and reliable 3D conformation, suitable for further computational procedures, such as molecular docking or molecular dynamics simulations. Muscaroside and muscariflavone derivatives were chosen for molecular docking due to their novelty, exclusive occurrence in *M. armeniacum*, and lack of previous comprehensive studies on their biological activities (Table S3). All ligands and proteins were prepared for docking using BIOVIA Discovery Studio and AutoDock v4.2.6.

### 4.15. Docking Grid and Parameters

The docking grid files were generated either from the literature or by using POCASA V1.1 (https://g6altair.sci.hokudai.ac.jp/g6/service/pocasa/, accessed on 18 May 2025): AChE (X: 31.062, Y: 20.311, Z: 11.947; 22 Å × 30 Å × 40 Å), BChE (X: 44.794, Y: −19.63, Z: −25.227; 30 Å × 30 Å × 30 Å), tyrosinase (X: −13.194, Y: 5.341, Z: −26.28; 26 Å × 26 Å × 28 Å), amylase (X: 14.188, Y: 48.964, Z: 22.886; 28 Å × 28 Å × 24 Å), and glucosidase (X: 3.091, Y: −8.008, Z: −4.08; 42 Å × 52 Å × 54 Å). Additionally, molecular docking was performed for LIHC targets, including, KEAP1 (X: −8.29, Y: 21.307, Z: 12.442; 60 Å × 74 Å × 40 Å), c-Myc (X: 71.779, Y: 68.731, Z: 41.5; 60 Å × 72 Å × 40 Å), YBX1 (X: 9.523, Y: 17.174, Z: 6.745; 52 Å × 40 Å × 46 Å), SMAD4 (X: 10.012, Y: −24.359, Z: −53.923; 58 Å × 80 Å × 54 Å), NF-p65 (X: 6.313, Y: 13.606, Z: −7.004; 30 Å × 22 Å × 24 Å), FOXM1 (X: 10.096, Y: 24.258, Z: −13.561; 28 Å × 28 Å × 28 Å), RAI_Pose-1 (X: 47.737, Y: 25.568, Z: 15.736; 40 Å × 40 Å × 40 Å), and ve RAI_Pose-2 (X: 46.932, Y: 6.236, Z: 51.181; 40 Å × 40 Å × 56 Å).

### 4.16. Validation and Interaction Analysis

Grid box sizes were determined based on the associated protein–ligand binding sites. AutoDock Vina V1.1.2 (https://vina.scripps.edu/ accessed on 18 May 2025) was used to search for different ligand conformations, with the exhaustiveness parameter set to 32. To verify the accuracy of protein docking, we redocked the proteins with their co-crystallized ligands and calculated the root mean square deviation (RMSD) values. To better understand the relationships between proteins, enzymes, and ligands, the scientists used the Protein Ligand Interaction Profiler (PLIP) (https://plip-tool.biotec.tu-dresden.de/plip-web/plip/index accessed on 18 May 2025), which provided deep insights into important interactions, such as hydrogen bonds (H-bonds). All compounds were visualized using PyMOL V2.5.8 and ChimeraX V1.7.1. These analytical methods provided evidence for the claims of credibility and accuracy of the docking results.

### 4.17. Molecular Dynamics Simulation

To investigate the stability and interactions of protein–ligand complexes, molecular dynamics (MD) simulations were performed using the CHARMM-GUI platform (https://charmm-gui.org, accessed on 18 May 2025). System setup was completed through the Solution Builder tool, following established methodologies. The CHARMM36m force field was applied to define protein parameters, ensuring accuracy in molecular behavior predictions. A solvation box containing TIP3P water molecules was generated, maintaining a 10 Å buffer around the protein. To achieve a physiological environment, counterions were introduced, and the NaCl concentration was adjusted to 0.15 M [53]. Electrostatic and van der Waals interactions were controlled using the Verlet cutoff method, while bond constraints were managed with the LINCS algorithm. Long-range electrostatics were computed using the particle mesh Ewald (PME) method. Prior to production runs, energy minimization was conducted with the steepest descent algorithm until the system’s potential energy fluctuations dropped below 1000 kJ/mol/nm. Following minimization, the system underwent equilibration under NVT and NPT ensembles at 310 K to ensure thermal stability [54]. The production phase involved 100 ns MD simulations (nstep = 50,000,000) executed in GROMACS 2023.3. The studied complexes included c-Myc_Muscaroside A, c-Myc_Muscaroside J, RAI_Pose2_Muscaroside A, RAI_Pose2_Muscaroside H, KEAP1_Muscariflavone A, KEAP1_Muscariflavone C, KEAP1_Muscaroside A, SMAD4_Pose2_Muscaroside G, and YBX1_Muscariflavone C, focusing on their conformational behavior and binding interactions. Post-simulation analyses encompassed RMSD, Root Mean Square Fluctuation (RMSF), Solvent-Accessible Surface Area (SASA), minimum distance calculations, and hydrogen bond profiling to assess the structural stability and interaction patterns of each complex. To further quantify the thermodynamic aspects of ligand binding, MM/PBSA (Molecular Mechanics/Poisson–Boltzmann Surface Area) calculations were conducted on the final 100 ns MD trajectories. Binding free energy (ΔG_bind) values were computed using the gmx_MMPBSA tool, enabling the decomposition of van der Waals, electrostatic, polar solvation, and nonpolar solvation contributions. The same nine complexes were subjected to this analysis, providing comparative insights into their binding affinities and interaction strengths.

### 4.18. Statistical Analysis

The mean values were calculated using three independent biological replicates. Statistical analysis was performed using GraphPad Prism 10 (GraphPad Software Inc., Boston, MA, USA). Statistically significant differences were determined by performing ANOVA, followed by Tukey’s test. Significance thresholds were set to *p* < 0.05.

## 5. Conclusions

We demonstrated different parts of *M*. *armeniacum* methanol and water extracts to examine their chemical profile and biological activities. The water extracts of flowers and leaves showed higher TPC, and the methanol extract of leaves showed higher TFC. The antioxidant activity varied greatly among the extracts used. The bulb methanolic and leaf water extracts showed higher antioxidant activity in this study. The results indicate that the bulbs are potential sources of phenolic compounds, and their methanolic extracts have good antioxidant activity. Similarly, the bulb methanolic extract showed potent AChE, BChE, and tyrosinase inhibition activity. The flower water extract, which had the highest phenolic content, led to the lowest expression of NRF2 and γ-GCS in HepG2 cells, suggesting that the phenolic compounds in the extract may have played a role in downregulating these genes. To complement these findings, in silico analysis highlighted muscaroside and muscariflavone derivatives as promising multi-target ligands, with stable interactions against AChE, BChE, KEAP1, c-Myc, and YBX1 supported by MD simulations and MM/PBSA calculations. Network pharmacology identified transcription factors such as YBX1, FOXM1, and RELA as key regulators of NRF2-related oxidative stress responses, further supporting the therapeutic potential of *M. armeniacum* extracts. These results provide mechanistic support for the experimental data and highlight multi-target therapeutic potential. Future studies should identify the specific phenolic compounds responsible for the antioxidant and enzyme inhibition activities of *M. armeniacum* extracts, particularly in the bulbs, and explore the molecular mechanisms by which the flower water extract downregulates NRF2 and γ-GCS expression and its potential impact on oxidative stress-related diseases.

## Figures and Tables

**Table 1:** Total phenolic and flavonoid contents in the tested extracts of *Muscari armeniacum* *.

Part	Extracts	TPC (mg GAE/g)	TFC (mg RE/g)
Flower	MeOH	18.44 ± 0.69 ^e^	7.26 ± 0.13 ^d^
	Water	26.47 ± 0.27 ^a^	10.61 ± 0.10 ^c^
Leaves	MeOH	22.44 ± 0.45 ^c^	27.30 ± 0.06 ^a^
	Water	23.76 ± 0.50 ^b^	20.94 ± 0.16 ^b^
Bulb	MeOH	20.82 ± 0.50 ^d^	3.44 ± 0.01 ^e^
	Water	5.64 ± 0.11 ^f^	0.95 ± 0.03 ^f^

* Values are reported as mean ± SD of three parallel measurements. GAE: Gallic acid equivalent; RE: Rutin equivalent. Different letters indicate significant differences between the tested extracts (*p* < 0.05).

**Table 2:** Chemical characterization of the tested extracts of *Muscari armeniacum*.

	Leaves		Flowers		Bulb	
Compounds	MeOH	Water	MeOH	Water	MeOH	Water
2,5-Dideoxy-2,5-imino-DL-glycero-D-manno-heptitol 7-*O*-apioside	+	−	−	−	+	−
2,5-Imino-2,5,6-trideoxy-DL-glycero-D-manno-heptitol	+	−	+	+	+	+
Hyacinthacine A1 or Hyacinthacine A2	+	+	+	+	+	+
2,5-Dideoxy-2,5-imino-DL-glycero-D-manno-heptitol	+	+	+	−	+	+
Hyacinthacine A3	+	−	+	+	+	−
Hyacinthacine B3	+	+	+	+	+	+
Hyacinthacine C1	+	+	+	+	+	+
2,5-Dideoxy-2,5-imino-DL-glycero-D-manno-heptitol 7-beta-D-xylopyranoside	+	−	+	−	+	+
Citric acid	+	+	+	+	+	+
Syringic acid-4-O-glucoside	+	+	+	+	+	−
Uralenneoside	+	+	+	+	+	−
Unidentified iridoid derivative 1	+	+	+	+	+	−
*p*-Coumaric acid-4-*O*-glucoside	+	+	+	+	−	−
Asperulosidic acid	+	+	+	+	+	−
Unidentified iridoid derivative 2	−	−	+	−	−	−
Ferulic acid-4-*O*-glucoside	+	+	−	−	−	−
1-O-(*p*-Coumaroyl)glucose trans isomer	−	−	+	+	−	−
*p*-Coumaric acid	+	−	+	+	−	−
Luteolin-*O*-trihexosylhexoside	+	+	+	−	−	−
Luteolin-O-dihexosylhexoside	+	+	+	−	−	−
*p*-Coumaroylmalic acid	−	−	+	−	−	−
Luteolin-O-(glucuronyl)pentosylhexoside	+	+	+	+	−	−
Muscariflavone A or isomer	+	+	+	+	+	+
Luteolin-7-O-sophoroside	+	+	−	−	−	−
Luteolin-O-glucuronylhexoside	+	+	+	+	−	−
Chrysoeriol-O-(glucuronyl)dipentosylhexoside isomer 1	+	+	+	+	−	−
Muscariflavone B	+	+	+	+	−	−
Muscariflavone C	+	+	+	+	−	−
Muscariflavone A or isomer	+	+	+	+	+	+
Chrysoeriol-O-(glucuronyl)dipentosylhexoside isomer 2	+	+	+	+	−	−
Chrysoeriol-O-(glucuronyl)pentosylhexoside	+	+	+	+	−	−
Apigenin-O-glucuronylhexoside	+	+	+	+	−	−
3′,5,7-Trihydroxyhomoisoflavanone	−	−	−	−	+	+
N-trans-Coumaroyltyramine	−	−	+	−	+	−
N-trans-Feruloyltyramine	−	−	+	−	+	−
Cosmosiin (Apigenin-7-O-glucoside)	+	+	−	−	−	−
Luteolin-O-(hydroxycinnamoyl)hexosylhexoside	+	+	−	−	−	−
5,8-Dihydroxy-3-(3,4-dihydroxybenzyl)-7-methoxy-4-chromanone	+	−	−	−	+	−
Apigenin-7-O-glucuronide	+	+	+	+	−	−
Chrysoeriol-O-glucuronide	+	+	+	+	−	−
Kaempferol-O-pentoside	−	−	+	+	−	−
Naringenin (4′,5,7-Trihydroxyflavanone)	−	−	+	+	+	−
7-Hydroxy-3-(3-hydroxy-4-methoxybenzyl)-5-methoxy-4-chromanone					+	+
5,7-Dihydroxy-3-(3,4-dihydroxybenzyl)-6-methoxy-4-chromanone	+	−	−	−	−	−
3′,4′,5,7-Tetrahydroxyhomoisoflavanone	+	−	−	−	+	+
Sacranoside A or isomer	+	+	+	+	−	−
Luteolin (3′,4′,5,7-Tetrahydroxyflavone)	+	−	+	−	−	−
Kaempferol-O-(*p*-coumaroyl)hexoside	−	−	+	+	−	−
Kaempferol (3,4′,5,7-Tetrahydroxyflavone)	−	−	+	−	−	−
5,7-Dihydroxy-3-(3-hydroxy-4-methoxybenzyl)-6-methoxy-4-chromanone	+	−	−	−	+	+
4′,5,7-Trihydroxyhomoisoflavanone	+	−	−	−	−	−
5,7-Dihydroxy-3-(4-hydroxy-3-methoxybenzyl)-6-methoxy-4-chromanone	+	−	−	−	+	+
7-Hydroxy-3-(3,4-dihydroxybenzyl)-5-methoxy-4-chromanone	−	−	−	−	+	+
7-Methoxy-3′,4′,5-trihydroxy-spiro[2H-1-benzopyran-3(4H),7′-bicyclo[4.2.0]octa[1,3,5]trien]-4-one	−	−	−	−	+	+
Apigenin (4′,5,7-Trihydroxyflavone)	+	+	+	+	+	−
Dihydroisomuscomosin	+	+			+	+
4′-Methoxy-3′,5,7-trihydroxy-spiro[2H-1-benzopyran-3(4H),7′-bicyclo[4.2.0]octa[1,3,5]trien]-4-one	−	−	−	−	+	−
Chrysoeriol (3′-Methoxy-4′,5,7-trihydroxyflavone)	+	+	+	+	−	−
Dimethoxy-trihydroxy(iso)flavone	+	−	+	+	−	−
Muscaroside G or isomer	+	+	+	+	+	+
Muscaroside B or E	+	+	+	+	+	+
Muscaroside H	+	+	+	+	+	+
Muscaroside A	+	+	+	+	+	+
Muscaroside J	+	−	+	+	+	−
Isomuscomosin	+	+	+	−	−	−
Muscaroside I	+	+	+	+	+	+
Phytosphingosine	−	−	−	−	+	−
Muscaroside C	+	+	+	−	+	+

+: present; −: absent.

**Table 3:** Antioxidant properties of the tested extracts of *Muscari armeniacum* *.

Part	Extracts	DPPH (mg TE/g)	ABTS (mg TE/g)	CUPRAC (mg TE/g)	FRAP (mg TE/g)	Chelating (mg EDTAE/g)	PBD (mmol TE/g)
Flower	MeOH	15.81 ± 0.64 ^d^	36.34 ± 1.04 ^c^	34.73 ± 1.59 ^d^	24.84 ± 0.80 ^c^	7.88 ± 0.37 ^e^	1.21 ± 0.11 ^a^
	Water	23.75 ± 0.26 ^c^	46.52 ± 0.68 ^b^	42.36 ± 0.60 ^c^	35.27 ± 0.51 ^b^	20.59 ± 0.40 ^d^	0.88 ± 0.03 ^b^
Leaves	MeOH	15.81 ± 0.29 ^d^	35.97 ± 0.09 ^c^	46.67 ± 0.82 ^b^	32.49 ± 0.53 ^b^	24.35 ± 0.27 ^b^	1.26 ± 0.05 ^a^
	Water	26.79 ± 0.09 ^b^	60.78 ± 0.35 ^a^	37.71 ± 1.85 ^d^	35.67 ± 0.90 ^b^	31.02 ± 0.11 ^a^	0.78 ± 0.05 ^b^
Bulb	MeOH	39.34 ± 0.31 ^a^	60.78 ± 0.56 ^a^	60.96 ± 1.33 ^a^	46.04 ± 3.29 ^a^	6.01 ± 0.39 ^f^	1.21 ± 0.02 ^a^
	Water	5.09 ± 0.44 ^e^	8.67 ± 1.18 ^d^	13.83 ± 0.14 ^e^	10.72 ± 0.18 ^d^	23.07 ± 0.28 ^c^	0.89 ± 0.01 ^b^

* Values are reported as mean ± SD of three parallel measurements. PBD: Phosphomolybdenum; MCA: Metal chelating activity; TE: Trolox equivalent; EDTAE: EDTA equivalent. Different letters indicate significant differences between the tested extracts (*p* < 0.05).

**Table 4:** Enzyme inhibitory properties of the tested extracts of *Muscari armeniacum* *.

Part	Extracts	AChE (mg GALAE/g)	BChE (mg GALAE/g)	Tyrosinase (mg KAE/g)	Amylase (mmol ACAE/g)	Glucosidase (mmol ACAE/g)
Flower	MeOH	1.82 ± 0.05 ^b^	1.26 ± 0.16 ^b^	42.36 ± 3.95 ^b^	0.51 ± 0.01 ^b^	0.02 ± 0.01 ^d^
	Water	0.21 ± 0.06 ^d^	na	na	0.14 ± 0.01 ^c^	0.11 ± 0.03 ^c^
Leaves	MeOH	1.70 ± 0.02 ^b^	1.46 ± 0.05 ^b^	44.68 ± 2.53 ^b^	0.57 ± 0.02 ^a^	0.16 ± 0.03 ^c^
	Water	0.41 ± 0.06 ^c^	na	na	0.10 ± 0.01 ^d^	0.27 ± 0.03 ^b^
Bulb	MeOH	1.96 ± 0.05 ^a^	2.19 ± 0.33 ^a^	57.19 ± 0.87 ^a^	0.56 ± 0.02 ^a^	2.32 ± 0.01 ^a^
	Water	0.11 ± 0.01 ^d^	na	na	0.07 ± 0.01 ^d^	na

* Values are reported as mean ± SD of three parallel measurements. GALAE: Galantamine equivalent; KAE: Kojic acid equivalent; ACAE: Acarbose equivalent; na: Not active. Different letters indicate significant differences between the tested extracts (*p* < 0.05).

## Data Availability

The authors confirm that the data supporting the findings of this study are available within the article and its Supplementary Materials.

## Supplementary Materials

The following supporting information can be downloaded at: https://www.mdpi.com/article/10.3390/molecules30132855/s1, Table S1: Primer sequences of genes analyzed in this study; Table S2: The docking score (kcal/mol) and interacting residues of the enzyme and protein. Table S3: Representations of selected bioactive compounds identified in *Muscari armeniacum* extracts and used for in silico analysis.
