# Supplementary material for: Integration Viewpoint Using UHPLC-MS/MS, In Silico Analysis, Network Pharmacology, and In Vitro Analysis to Evaluate the Bio-Potential of Muscari armeniacum Extracts
Source: Molecules. 2025 Jul 4;30(13):2855. doi: 10.3390/molecules30132855 (PMC12250750; doi:10.3390/molecules30132855)
Supplement: Supplementary file 1 [file molecules-30-02855-s001.zip › molecules-3702500-supplementary.pdf]

Integration Viewpoint Using UHPLC-MS/MS, In Silico Analysis, Network Pharmacology, and In Vitro Analysis to Evaluate the Bio-Potential of *Muscari armeniacum* Extracts

Nilofar [1,2], Gokhan Zengin[1*], Mehmet Veysi Cetiz[3], Evren Yildiztugay[4], Zoltán Cziáky[5], József Jeko[5], Claudio Ferrante[2], Tina Kostka[6,7], Tuba Esatbeyoglu[6], Stefano Dall'Acqua[8*]

[1]*Department of Biology, Science Faculty, Selcuk University, 42130 Konya, Türkiye*, nelofarkhattak@gmail.com (NN) gokhanzengin@selcuk.edu.tr (GZ)

[2]*Department of Pharmacy, Botanic Garden "Giardino dei Semplici", Università degli Studi "Gabriele d'Annunzio", via dei Vestini 31, 66100 Chieti, Italy*, claudio.ferrante@unich.it (CF)

[3]*Department of Medical Biochemistry, Faculty of Medicine, Harran University, Sanliurfa, 63290, Turkey*, mvcetiz@gmail.com (MVC)

[4]*Department of Biotechnology, Science Faculty, Selcuk University, 42130 Konya, Türkiye*, eytugay@gmail.com (EY)

[5]*Agricultural and Molecular Research and Service Institute, University of Nyíregyháza, 001, Nyíregyháza, Hungary*, cziaky.zoltan@nye.hu (CZ), jjozsi@gmail.com (JJ)

[6]*Department of Molecular Food Chemistry and Food Development, Institute of Food and One Health, Gottfried Wilhelm Leibniz University Hannover, Am Kleinen Felde 30, 30167 Hannover, Germany*, kostka@chemie.uni-kl.de (TK), esatbeyoglu@foh.uni-hannover.de (TB)

[7]*Division of Food Chemistry and Toxicology, Department of Chemistry, RPTU Kaiserslautern-Landau, Erwin-Schrödinger-Strasse 52, 67663 Kaiserslautern, Germany*

[8]*Department of Pharmaceutical and Pharmacological Sciences, University of Padova, 35131, Padua, Italy*, stefano.dallacqua@unipd.it (SD)

* Correspondence: gokhanzengin@selcuk.edu.tr; stefano.dallacqua@unipd.it

UHPLC-MS/MS metabolomic analysis

The phytochemical analysis of leaves, flower and bulb extracts *Muscari armeniacum* was performed using ultra high-performance liquid chromatography (UHPLC) coupled with an electrospray ionisation source and an Orbitrap-based high-resolution, accurate-mass (HRAM) performance MS system (Thermo Q-Exactive, Thermo Scientific, USA). A previously developed and successfully applied gradient UHPLC separation was carried out on a Thermo Accucore analytical C18 column (100 mm x 2.1, mm i. d., 2.6 μm) using a Dionex 3000RS UltiMate system. Analysis performed with the following parameters provided optimal peak symmetry, good resolution, and significantly enhanced sensitivity: column temperature was set at 25 °C ± 1 °C. Mobile phase consisted of water (A) and methanol (B). Both was acidified with 0.1 % formic acid. The solvent flow rate and injection volume was set at 0.2 mL/min and as 2 μL, respectively. The sample solutions were filtered through 0.22 μm filters. The total duration of the gradient was 70 min length with a 3 min isocratic segment (5% solvent B), a linear gradient increasing from 5% B to 100% (3-43 min), 100% B (43-61 min), a linear gradient decreasing from 100% B to 5% (61-62 min) and 5 % B (62-70 min). ESI spectra were recorded in both positive and negative ionization modes. All LC–MS/MS data acquisitions were performed and monitored using Xcalibur 3.0.63 software (Thermo Scientific Inc, Waltham, MA, USA). All data were processed using Thermo TraceFinder software and tentatively identified by comparing their data (retention time, fragmentation pattern) with our spectral library and online databases (Massbank of North America, m/z Cloud). The difference between the measured and calculated monoisotopic molecular masses was less than 5 ppm in every case [1].

Table S1: Primer sequences of genes analyzed in this study.

Reference Gene	Sequence (5’-3’)
GAPDH forward	AGCCACATCGCTCAGACAC
GAPDH reverse	GCCCAATACGACCAAATCC
Genes involved in the redox system	
HMOX1 forward	CCAGGCAGAGAATGCTGAGT
HMOX1 reverse	GTAGACAGGGGCGAAGACTG
NFE2L2 forward	ACACGGTCCACAGCTCATC
NFE2L2 reverse	TGTCAATCAAATCCATGTCCTG
GCLC forward	TTTGGTCAGGGAGTTTCCAG
GCLC reverse	TGAACAGGCCATGTCAACTG

Table S2. The docking score (kcal/mol) and interacting residues of the enzyme and protein.

Compound	Target	PDB ID	Binding energy	RMSD	Interaction		Binding site
					Type	Number	
Muscaroside A	TYR	5M8O	-8.7	7.6509	Hbond	9	LYS A:198;VAL A:211;ASN A:318;ARG A:321;ARG A:321;ARG A:374;ASN A:378;GLN A:390;THR A:391
Muscaroside H	TYR	5M8O	-9.1	0.5695	Hbond	14	VAL A:196;ASN A:318;ASN A:318;ARG A:321;ARG A:321;ARG A:321;ARG A:374;ARG A:374;LEU A:382;ASN A:385;ASN A:385;ASN A:385;THR A:391;THR A:391
Muscariflavone A	TYR	5M8O	-9.6	0.5264	Hbond	22	HIS A:192;LYS A:197;GLY A:209;VAL A:211;ASP A:212;HIS A:215;ARG A:321;ARG A:321;ARG A:374;ARG A:374;LEU A:382;ASN A:385;ASN A:385;GLY A:386;GLY A:386;GLN A:390;THR A:391;THR A:391;THR A:391;HIS A:392;HIS A:392;SER A:394
Muscaroside G	TYR	5M8O	-8.6	nd	Hbond	15	ASN A:318;ASN A:318;ALA A:320;ARG A:321;ARG A:321;ARG A:321;ARG A:374;ARG A:374;ASN A:385;ASN A:385;GLY A:386;GLN A:390;THR A:391;THR A:391;HIS A:392
Muscariflavone B	TYR	5M8O	-9.5	0.2703	Hbond	14	HIS A:192;HIS A:215;GLU A:216;ASN A:318;ARG A:321;ARG A:321;ARG A:374;ARG A:374;ASN A:385;ASN A:385;GLN A:390;THR A:391;HIS A:392;SER A:394
Muscariflavone C	TYR	5M8O	-8.3	0.5756	Hbond	11	HIS A:215;ASN A:318;ARG A:321;ARG A:321;ARG A:321;ARG A:374;ARG A:374;HIS A:377;THR A:391;THR A:391;HIS A:392
Muscaroside J	TYR	5M8O	-9.6	0.884	Hbond	10	HIS A:215;TYR A:362;ARG A:374;ARG A:374;ASN A:378;HIS A:381;GLY A:389;GLN A:390;THR A:391;HIS A:392
Muscaroside C	TYR	5M8O	-8.5	0.0161	Hbond	14	VAL A:196;LYS A:198;HIS A:215;ARG A:321;ARG A:321;TYR A:362;TYR A:362;ASN A:378;HIS A:381;THR A:391;THR A:391;HIS A:392;HIS A:392;SER A:394
Muscaroside I	BChE	3DJY	-9.6	1.098	Hbond	9	ASN A:68;GLN A:71;SER A:72;GLN A:119;GLU A:276;GLU A:276;GLY A:283;THR A:284;ASN A:289
Muscaroside A	BChE	3DJY	-11.6	0.4502	Hbond	15	ASP A:70;GLN A:71;GLY A:115;GLY A:115;GLY A:116;THR A:122;TYR A:128;TYR A:128;GLU A:197;ALA A:199;SER A:287;SER A:287;TYR A:332;TYR A:332;HIS A:438
Muscaroside B	BChE	3DJY	-11.2	0.9983	Hbond	20	ASN A:68;ILE A:69;GLN A:71;SER A:72;GLY A:78;TRP A:82;GLY A:115;GLY A:115;GLY A:117;THR A:120;TYR A:128;ALA A:199;ALA A:199;LEU A:273;PRO A:285;SER A:287;ASN A:289;ASN A:289;TRP A:430;HIS A:438
Muscaroside H	BChE	3DJY	-10.5	6.7755	Hbond	16	GLN A:67;ASN A:68;ASP A:70;ASP A:70;ASP A:70;ASP A:70;SER A:72;GLY A:78;GLU A:276;GLU A:276;THR A:284;LEU A:286;LEU A:286;ASN A:289;TYR A:332;HIS A:438
Muscariflavone A	BChE	3DJY	-11.3	0.9284	Hbond	17	ASN A:68;ILE A:69;GLN A:71;SER A:72;ASN A:83;GLY A:115;GLY A:115;GLY A:115;GLY A:117;GLN A:119;THR A:120;TYR A:128;ALA A:199;GLU A:276;PRO A:285;LEU A:286;HIS A:438
Muscaroside G	BChE	3DJY	-11.0	8.9133	Hbond	17	ASP A:70;ASP A:70;GLY A:115;GLN A:119;LEU A:274;GLU A:276;GLU A:276;ALA A:277;PRO A:281;GLY A:283;GLY A:283;PRO A:285;SER A:287;ASN A:289;ASN A:289;ASN A:289;TYR A:332
Muscariflavone B	BChE	3DJY	-10.9	0.6525	Hbond	15	GLN A:67;ASP A:70;ASN A:83;GLY A:116;GLY A:117;ALA A:199;ALA A:199;GLU A:276;PRO A:285;ASN A:289;TYR A:332;HIS A:438;HIS A:438;TYR A:440;TYR A:440
Muscariflavone C	BChE	3DJY	-10.7	0.947	Hbond	10	ASP A:70;TRP A:82;GLY A:116;GLY A:117;GLY A:117;GLU A:197;GLU A:197;ALA A:199;SER A:287;HIS A:438
Muscaroside J	BChE	3DJY	-11.0	0.75	Hbond	8	ASP A:70;ALA A:277;VAL A:280;GLY A:283;SER A:287;ASN A:289;ASN A:289;ASN A:289
Muscaroside C	BChE	3DJY	-10.2	1.0564	Hbond	15	ASN A:68;ILE A:69;GLN A:71;SER A:72;SER A:72;GLY A:115;GLU A:276;GLU A:276;ALA A:277;THR A:284;PRO A:285;SER A:287;ASN A:289;ASN A:289;ASN A:289
**Muscaroside A**	Glucosidase	3W37	-8.4	1.8753	Hbond	11	ASP A:232;ASP A:232;SER A:497;SER A:497;SER A:497;SER A:497;ARG A:552;ARG A:552;ASP A:568;ASP A:568;ASP A:568
**Muscaroside H**	Glucosidase	3W37	-8.6	1.0382	Hbond	11	ASP A:232;ASP A:232;PHE A:476;SER A:497;SER A:497;SER A:505;ARG A:552;ARG A:552;ASP A:568;ASP A:568;GLU A:603
**Muscariflavone A**	Glucosidase	3W37	-9.7	0.4948	Hbond	10	ASP A:232;ASP A:232;ALA A:234;ASN A:237;ASP A:357;SER A:497;SER A:497;SER A:505;LYS A:506;HIS A:626

**Muscaroside G**	Glucosidase	3W37	-8.5	4.7754	Hbond	17	ASP A:232;ASP A:232;ALA A:234;ASN A:237;ASN A:237;ASN A:475;PHE A:476;SER A:497;SER A:497;SER A:497;SER A:505;SER A:505;ARG A:552;ASP A:568;ARG A:572;ARG A:572;GLU A:603
**Muscariflavone B**	Glucosidase	3W37	-9.5	0.1315	Hbond	15	ASP A:232;ALA A:234;ASN A:237;ASN A:237;ASP A:357;ASN A:496;ASN A:496;SER A:497;SER A:497;SER A:497;SER A:497;SER A:505;SER A:505;LYS A:506;HIS A:626
**Muscariflavone C**	Glucosidase	3W37	-9.2	0.3455	Hbond	15	ASP A:232;ASP A:232;ALA A:234;ASN A:237;PHE A:476;ASN A:496;SER A:497;SER A:497;SER A:497;SER A:497;SER A:497;SER A:505;LYS A:506;LYS A:506;ASP A:630
**Muscaroside J**	Glucosidase	3W37	-8.0	7.5899	Hbond	8	ASP A:232;ASP A:232;ASP A:232;ASN A:496;SER A:497;SER A:497;SER A:505;ASP A:568
**Muscaroside A**	AChE	2Y2V	-10.5	0.1956	Hbond	14	TYR A:72;ASP A:74;THR A:75;THR A:75;LEU A:76;ASN A:87;GLY A:121;GLY A:122;TYR A:124;TYR A:124;GLU A:202;PHE A:295;TYR A:341;HIS A:447
**Muscaroside B**	AChE	2Y2V	-9.4	13.1112	Hbond	19	TYR A:72;TYR A:72;THR A:75;ASN A:87;GLY A:122;TYR A:124;TYR A:133;TYR A:133;GLU A:202;GLN A:291;SER A:293;PHE A:295;ARG A:296;ARG A:296;ARG A:296;TYR A:337;TYR A:341;TYR A:341;HIS A:447
**Muscariflavone A**	AChE	2Y2V	-10.2	0.8271	Hbond	8	TYR A:72;THR A:75;THR A:75;LEU A:76;SER A:293;SER A:293;PHE A:295;VAL A:340
**Muscariflavone B**	AChE	2Y2V	-9.9	0.8787	Hbond	17	TYR A:72;TYR A:72;THR A:75;LEU A:76;GLY A:120;GLY A:121;TYR A:124;SER A:125;GLU A:202;GLU A:202;ALA A:204;GLU A:292;PHE A:295;ARG A:296;ARG A:296;TYR A:341;HIS A:447
**Muscariflavone C**	AChE	2Y2V	-9.8	0.9595	Hbond	10	TYR A:72;TYR A:72;ASP A:74;TYR A:77;GLY A:120;TYR A:124;TYR A:133;TYR A:133;TRP A:286;TYR A:341
**Muscaroside J**	AChE	2Y2V	-10.5	0.9927	Hbond	8	GLY A:121;TYR A:124;TYR A:124;SER A:125;GLU A:202;PHE A:295;TYR A:341;HIS A:447
**Muscaroside C**	AChE	2Y2V	-9.2	1.0483	Hbond	10	TYR A:72;ASP A:74;GLY A:121;GLY A:122;TYR A:124;GLU A:202;GLU A:202;ALA A:204;PHE A:295;ARG A:296
**Muscaroside I**	Amylase	2QV4	-8.4	0.771	Hbond	12	ILE A:148;ILE A:148;TYR A:151;THR A:163;ALA A:198;LYS A:200;HIS A:201;GLU A:233;GLU A:240;ASP A:300;GLY A:304;HIS A:305
**Muscaroside A**	Amylase	2QV4	-10.6	4.8295	Hbond	8	ASN A:105;ILE A:148;TYR A:151;THR A:163;GLU A:233;ASP A:300;HIS A:305;HIS A:305
**Muscaroside B**	Amylase	2QV4	-8.4	13.1112	Hbond	9	ALA A:198;LYS A:200;LYS A:200;GLU A:233;ILE A:235;ILE A:235;HIS A:299;HIS A:305;HIS A:305
**Muscaroside H**	Amylase	2QV4	-9.0	0.6409	Hbond	8	ASN A:105;ILE A:148;THR A:163;LYS A:200;HIS A:201;GLU A:233;ASP A:300;GLY A:304
**Muscariflavone A**	Amylase	2QV4	-9.4	0.3334	Hbond	7	TRP A:59;GLN A:63;ILE A:148;ARG A:195;HIS A:201;GLU A:233;HIS A:299
**Muscaroside G**	Amylase	2QV4	-9.5	7.885	Hbond	10	ASN A:105;ALA A:106;TYR A:151;LYS A:200;HIS A:201;GLU A:233;GLY A:304;HIS A:305;GLY A:306;GLY A:308
**Muscariflavone B**	Amylase	2QV4	-9.6	1.0647	Hbond	11	GLY A:104;ALA A:106;TYR A:151;ARG A:195;ASP A:197;ALA A:198;LYS A:200;LYS A:200;ILE A:235;HIS A:299;HIS A:305
**Muscariflavone C**	Amylase	2QV4	-9.4	0.3739	Hbond	12	ASN A:53;TYR A:62;HIS A:101;ALA A:106;VAL A:107;TYR A:151;THR A:163;ARG A:195;ASP A:197;HIS A:201;ASP A:300;HIS A:305
**Muscaroside J**	Amylase	2QV4	-10.3	0.0936	Hbond	5	TYR A:151;TYR A:151;ARG A:195;ALA A:198;HIS A:305
**Muscaroside C**	Amylase	2QV4	-8.7	0.6258	Hbond	9	ASN A:105;THR A:163;ALA A:198;LYS A:200;HIS A:201;GLU A:233;ASP A:300;ASP A:300;HIS A:305
**Muscariflavone A**	YBX1	6KTC	-8.9	0.8753	Hbond	8	TYR A:22;TYR A:22;HIS A:37;THR A:39;ARG A:51;LYS A:68;ASN A:74;ASN A:74
**Muscariflavone C**	YBX1	6KTC	-8.2	0.495	Hbond	12	ASN A:20;ASP A:33;ASP A:33;ASP A:33;ASP A:33;HIS A:37;GLY A:66;LYS A:68;GLY A:69;GLY A:69;GLU A:71;ASN A:74
**Muscariflavone A**	SMAD4_Pose-1	1DD1	-8.1	nd	Hbond	9	ILE A:314;SER A:343;GLU A:394;ASP A:396;LYS A:436;ARG A:441;ARG A:441;ARG A:441;ARG A:531
Muscaroside G	SMAD4_Pose-2	1DD1	-8.0	1.0178	Hbond	12	LYS A:392;ARG A:400;ARG A:400;CYS A:401;LEU A:402;TYR A:413;SER A:432;SER A:432;TYR A:434;TYR A:434;TYR A:434;LYS A:436

**Muscaroside A**	c-Myc	1NKP	-8.5	0.2437	Hbond	8	ARG A:925;ARG A:925;ARG A:925;ASP A:926;GLU A:932;ASN A:934;GLU A:935;LYS A:939
**Muscariflavone A**	c-Myc	1NKP	-8.0	0.067	Hbond	10	ARG A:925;ASP A:926;GLN A:927;GLN A:927;LEU A:931;GLU A:932;ASN A:934;GLU A:935;TYR A:949;TYR A:949
**Muscaroside J**	c-Myc	1NKP	-8.2	1.0939	Hbond	8	ARG A:925;ARG A:925;ILE A:928;ILE A:928;GLU A:932;ASN A:933;GLU A:935;LYS A:939
**Muscaroside A**	KEAP1	6T7V	-10.4	0.5439	Hbond	14	ARG A:326;VAL A:418;VAL A:465;VAL A:467;VAL A:467;ARG A:470;VAL A:512;HIS A:516;ILE A:559;THR A:560;THR A:560;VAL A:606;VAL A:608;VAL A:608
**Muscaroside B**	KEAP1	6T7V	-9.4	0.1986	Hbond	18	ARG A:326;GLY A:367;VAL A:369;VAL A:369;GLY A:371;GLY A:372;VAL A:418;VAL A:420;VAL A:420;ARG A:470;VAL A:514;HIS A:516;ILE A:559;THR A:560;VAL A:606;VAL A:606;THR A:609;THR A:609
**Muscaroside H**	KEAP1	6T7V	-9.7	0.8716	Hbond	14	VAL A:369;GLY A:371;VAL A:420;ASP A:422;ASP A:422;ARG A:470;VAL A:512;VAL A:512;HIS A:516;ILE A:559;THR A:560;VAL A:561;VAL A:561;VAL A:608
**Muscariflavone A**	KEAP1	6T7V	-11.4	1.1593	Hbond	17	ARG A:415;ARG A:415;VAL A:418;VAL A:418;VAL A:420;VAL A:465;ALA A:466;VAL A:467;ASN A:469;ALA A:556;ILE A:559;THR A:560;THR A:560;VAL A:604;VAL A:606;VAL A:606;VAL A:608
**Muscaroside G**	KEAP1	6T7V	-9.0	5.8899	Hbond	14	GLY A:367;VAL A:369;VAL A:369;VAL A:418;VAL A:420;VAL A:420;LEU A:515;LEU A:515;HIS A:516;ILE A:559;THR A:560;VAL A:561;GLY A:564;VAL A:608
**Muscariflavone B**	KEAP1	6T7V	-10.4	0.915	Hbond	14	ARG A:326;ARG A:326;LEU A:365;LEU A:365;VAL A:418;VAL A:418;VAL A:465;VAL A:465;ASN A:469;ILE A:559;THR A:560;VAL A:606;VAL A:606;VAL A:608
**Muscariflavone C**	KEAP1	6T7V	-11.5	1.0045	Hbond	19	ARG A:326;ILE A:416;ILE A:416;VAL A:418;VAL A:465;ALA A:466;ARG A:470;ALA A:510;VAL A:512;VAL A:512;VAL A:514;HIS A:516;ILE A:559;THR A:560;VAL A:561;VAL A:604;VAL A:606;VAL A:606;VAL A:608
**Muscaroside J**	KEAP1	6T7V	-10.1	5.8415	Hbond	13	ARG A:326;VAL A:418;VAL A:463;VAL A:465;VAL A:465;VAL A:465;VAL A:467;ILE A:559;ILE A:559;THR A:560;THR A:560;THR A:560;VAL A:606
**Muscaroside C**	KEAP1	6T7V	-8.5	0.9034	Hbond	7	ARG A:326;VAL A:369;VAL A:369;VAL A:420;ASP A:422;GLY A:423;VAL A:606
Muscaroside A	RAI_Pose-1	2VGE	-8.2	0.2175	Hbond	5	ASN A:657;ASN A:666;ASP A:690;HIS A:692;ARG A:738
Muscaroside H	RAI_Pose-1	2VGE	-8.3	1.0184	Hbond	6	ASP A:633;ASP A:633;ASN A:666;ASN A:666;SER A:691;HIS A:692
**Muscaroside A**	RAI_Pose-2	2VGE	-8.3	0.8636	Hbond	9	THR A:722;GLU A:776;GLU A:776;ASP A:797;ASP A:797;ASP A:797;TRP A:798;ARG A:812;ASN A:813
**Muscaroside B**	RAI_Pose-2	2VGE	-8.0	nd	Hbond	8	ASN A:685;THR A:722;THR A:722;THR A:722;THR A:722;GLU A:772;TRP A:798;ASN A:813
**Muscaroside H**	RAI_Pose-2	2VGE	-9.3	0.6073	Hbond	12	PHE A:720;THR A:729;GLN A:753;GLN A:753;ARG A:790;ASP A:791;ASP A:797;ARG A:812;ARG A:812;ARG A:812;ARG A:812;ASN A:813
**Muscariflavone A**	RAI_Pose-2	2VGE	-8.5	1.0876	Hbond	9	ASP A:652;ASP A:652;ASN A:685;GLY A:693;THR A:722;TRP A:767;ASP A:768;ARG A:820;LYS A:822
**Muscaroside G**	RAI_Pose-2	2VGE	-8.7	15.8152	Hbond	15	THR A:722;THR A:729;GLU A:752;ARG A:790;GLU A:795;THR A:796;THR A:796;ASP A:797;TRP A:799;ARG A:812;ARG A:812;ARG A:812;ARG A:812;ASN A:813;ASN A:813
**Muscaroside J**	RAI_Pose-2	2VGE	-8.3	0.2565	Hbond	14	ASN A:685;PHE A:720;THR A:722;THR A:722;THR A:722;THR A:722;TRP A:767;ASP A:797;ASP A:797;ASP A:797;ARG A:812;ARG A:812;ASN A:813;ASN A:813
**Muscaroside C**	RAI_Pose-2	2VGE	-8.2	0.9119	Hbond	11	THR A:722;ASP A:797;ASP A:797;TRP A:798;ARG A:812;ARG A:812;ARG A:812;ASN A:813;ASN A:813;ASN A:813;TYR A:814

Table S3: Representations of selected bioactive compounds identified in *Muscari armeniacum* extracts and used for in silico analysis.

Compound	Smiles	Structure
Muscaroside I	CCC(=O)[C@@H]1C[C@@H](C)[C@]2(CC[C@@]3(C)C4=C(CC[C@]23C)[C@@]5(C)CC[C@H](O[C@@H]6O[C@H](CO[C@@H]7OC[C@H](O)[C@H](O)[C@H]7O[C@@H]8O[C@H](CO)[C@@H](O)[C@H](O[C@@H]9O[C@H](CO)[C@H](O)[C@H](O)[C@H]9O[C@@H]%10O[C@@H](CO)[C@H](O)[C@H]%10O)[C@H]8O[C@@H]%11OC[C@](O)(CO)[C@H]%11O)[C@@H](O)[C@H](O)[C@H]6O)C(CO)(CO)[C@@H]5CC4)O1	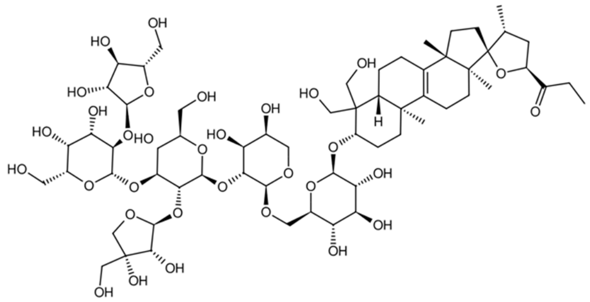
Hyacinthacine C1	C[C@@H]1[C@@H](O)[C@H](O)[C@@H]2[C@H](O)[C@H](O)[C@@H](CO)N21	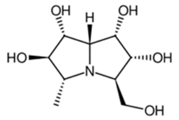
Muscaroside A	CCC(=O)[C@@H]1C[C@@H](C)[C@]2(CC(=O)[C@@]3(C)C4=C(CC[C@]23C)[C@@]5(C)CC[C@H](O[C@@H]6O[C@H](CO[C@@H]7OC[C@H](O)[C@H](O)[C@H]7O[C@@H]8O[C@H](CO)[C@@H](O)[C@H](O)[C@H]8O[C@@H]9O[C@@H](C)[C@H](O)[C@@H](O)[C@H]9O)[C@@H](O)[C@H](O)[C@H]6O)[C@](C)(CO)[C@@H]5CC4)O1	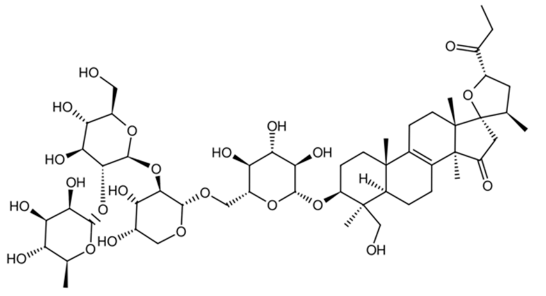

Muscaroside B	CCC(=O)C1C[C@@H](C)[C@]2(CC(=O)[C@@]3(C)C4=C(CC[C@@]32C)[C@@]2(C)CC[C@H](O[C@@H]3O[C@H](CO[C@@H]5OC[C@H](O)[C@H](O)[C@H]5O[C@@H]5O[C@H](CO)[C@@H](O)[C@H](O[C@@H]6OC[C@H](O)[C@H](O)[C@H]6O[C@@H]6O[C@@H](CO)[C@H](O)[C@@H]6O)[C@H]5O[C@@H]5O[C@@H](C)[C@H](O)[C@@H](O)[C@H]5O)[C@@H](O)[C@H](O)[C@H]3O)[C@](C)(CO)[C@@H]2CC4)O1	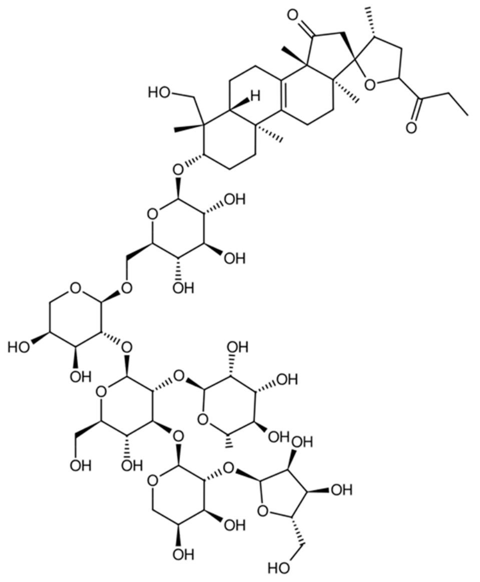

Muscaroside H	CCC(=O)[C@@H]1C[C@@H](C)[C@]2(CC(=O)[C@@]3(C)C4=C(CC[C@]23C)[C@@]5(C)CC[C@H](O[C@@H]6O[C@H](CO[C@@H]7OC[C@H](O)[C@H](O)[C@H]7O[C@@H]8O[C@H](CO)[C@@H](O)[C@H](O[C@@H]9OC[C@H](O)[C@H](O)[C@H]9O)[C@H]8O[C@@H]%10O[C@@H](C)[C@H](O)[C@@H](O)[C@H]%10O)[C@@H](O)[C@H](O)[C@H]6O)[C@](C)(CO)[C@@H]5CC4)O1	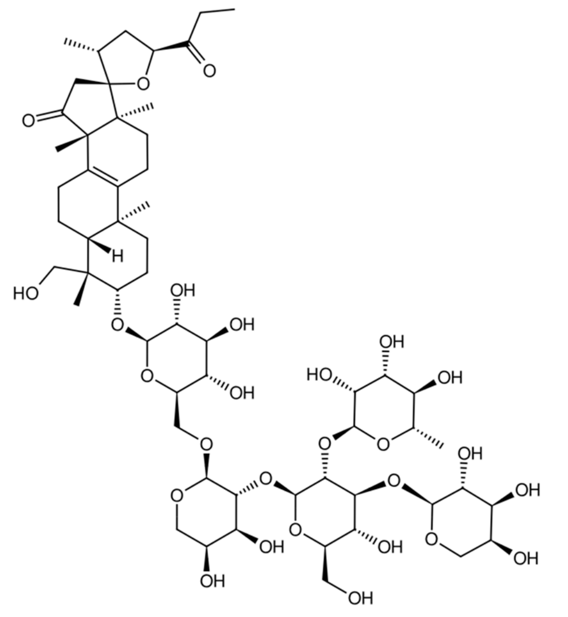
Muscariflavone A	C1=CC=C(C=C1)C2=CC(=O)C3=C(O2)C=C(C=C3O)O[C@H]4O[C@@H](CO)[C@H](O)[C@@H](O)[C@H](O[C@H]5O[C@@H](CO)[C@H](O)[C@@H](O)[C@H](O[C@H]6O[C@@H](CO)[C@H](O)[C@H](O)[C@@H](O6))C5)C4	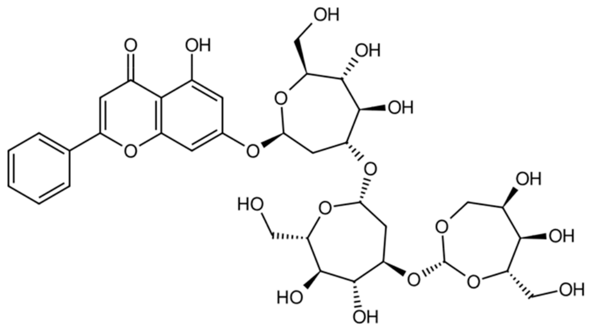

Muscaroside G	CCC(=O)[C@@H]1C[C@@H](C)[C@]2(CC(=O)[C@@]3(C)C4=C(CC[C@]23C)[C@@]2(C)CC[C@@H]([C@](C)(CO)[C@@H]2CC4)O[C@H]2[C@@H]([C@H]([C@@H]([C@@H](CO[C@H]3[C@@H]([C@H]([C@H](CO3)O)O)O[C@H]3[C@@H]([C@H]([C@@H]([C@@H](CO)O3)O)O[C@H]3[C@@H]([C@H]([C@H](CO3)O)O)O[C@@H]3[C@H]([C@@H]([C@H]([C@H](CO)O3)O)O)O)O[C@H]3[C@@H]([C@@H]([C@H]([C@H](C)O3)O)O)O)O2)O)O)O)O1	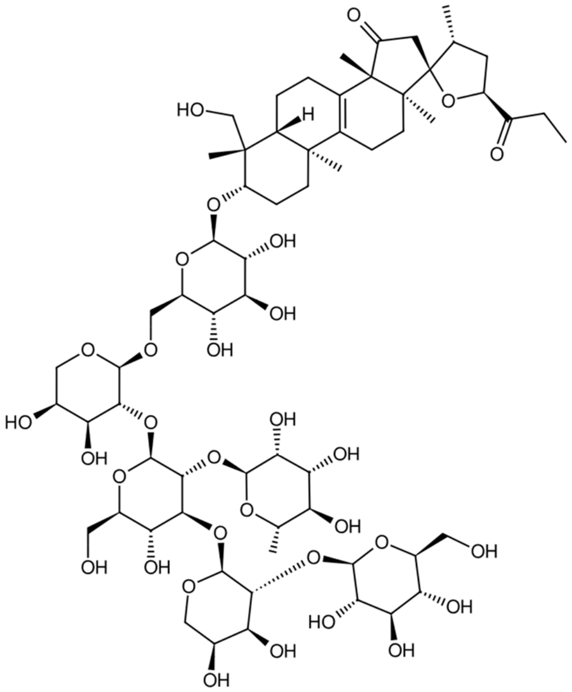
Hyacinthacine A3	C[C@H]1CC[C@H]2[C@@H](O)[C@H](O)[C@@H](CO)N12	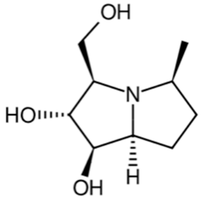

Muscariflavone B	C1=CC=C(C=C1)C2=CC(=O)C3=C(C=C(C=C3O2)O)O[C@H]4O[C@@H](CO)[C@H](O)[C@@H](O)[C@H](O[C@H]5O[C@@H](CO)[C@H](O)[C@@H](O)[C@H](O[C@H]6O[C@@H](CO)[C@H](O)[C@@H](O)[C@H](O6))C5)C4	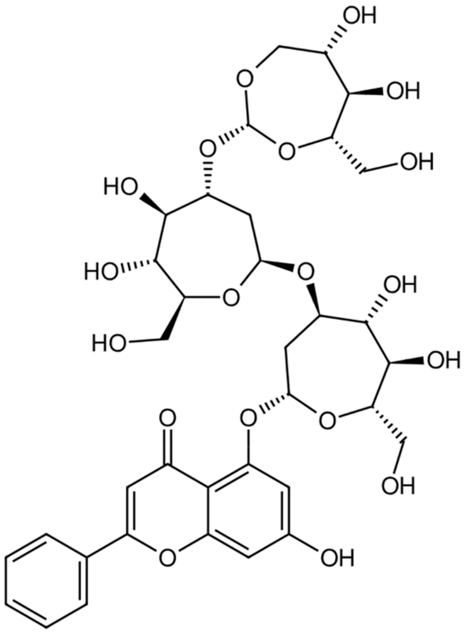
Hyacinthacine B3	C[C@@H]1C[C@H]([C@H]2N1[C@@H]([C@H]([C@H]2O)O)CO)O	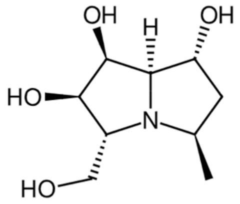

Muscariflavone C	C1=CC=C(C=C1)C2=CC(=O)C3=C(C=C(C=C3O2)O)O[C@H]4O[C@@H](CO)[C@H](O)[C@@H](O)[C@H](O[C@H]5O[C@@H](CO)[C@H](O)[C@@H](O)[C@H](O[C@H]6O[C@@H](CO)[C@H](O)[C@@H](O)[C@H](O[C@H]7O[C@@H](CO)[C@H](O)[C@H](O)[C@@H](O7))C6)C5)C4	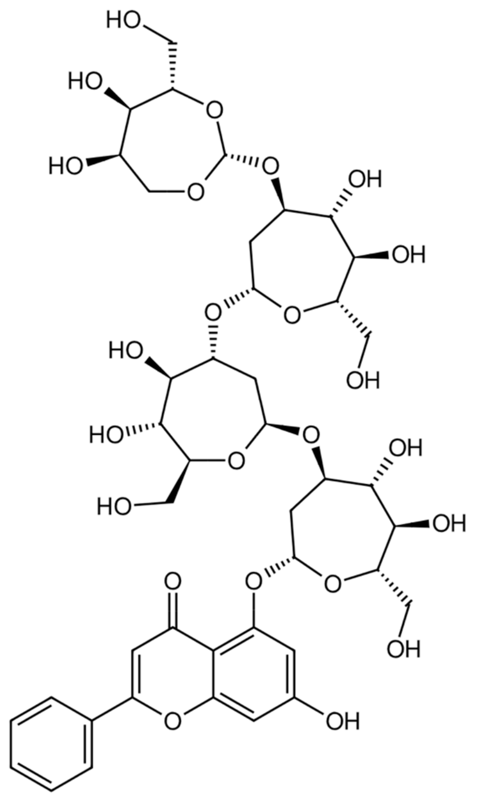

Muscaroside J	CCC(=O)[C@@H]1C[C@@H](C)[C@]2(CC(=O)[C@@]3(C)C4=C(CC[C@]23C)[C@@]5(C)CC[C@H](O[C@@H]6O[C@H](CO[C@@H]7OC[C@H](O)[C@H](O)[C@H]7O[C@@H]8O[C@H](CO)[C@@H](O)[C@H](O)[C@H]8O)[C@@H](O)[C@H](O)[C@H]6O)[C@](C)(CO)[C@@H]5CC4)O1	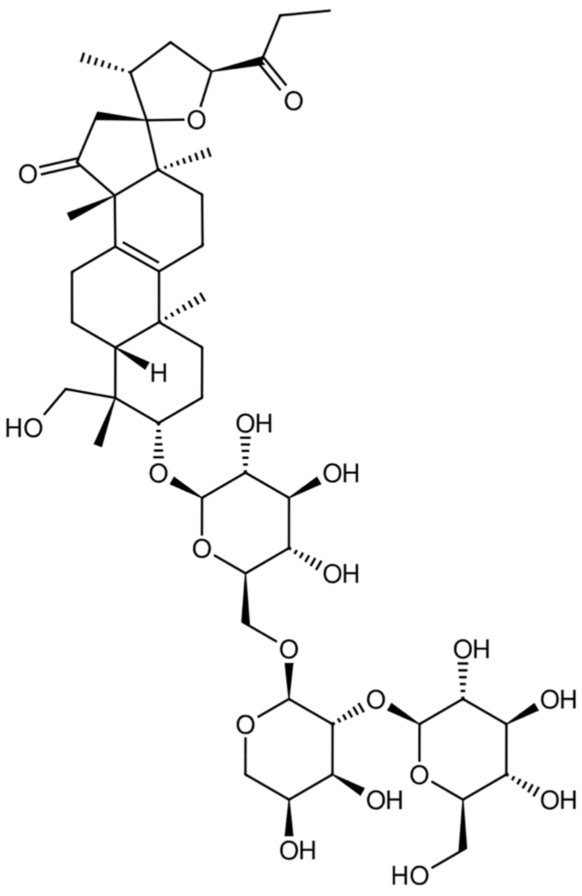

Muscaroside C	CCC(=O)[C@@H]1C[C@@H](C)[C@]2(CC[C@@]3(C)C4=C(CC[C@]23C)[C@@]5(C)CC[C@H](O[C@@H]6O[C@H](CO[C@@H]7OC[C@H](O)[C@H](O)[C@H]7O[C@@H]8O[C@H](CO)[C@@H](O)[C@H](O)[C@H]8O[C@@H]9OC[C@](O)(CO)[C@H]9O)[C@@H](O)[C@H](O)[C@H]6O)C(CO)(CO)[C@@H]5CC4)O1	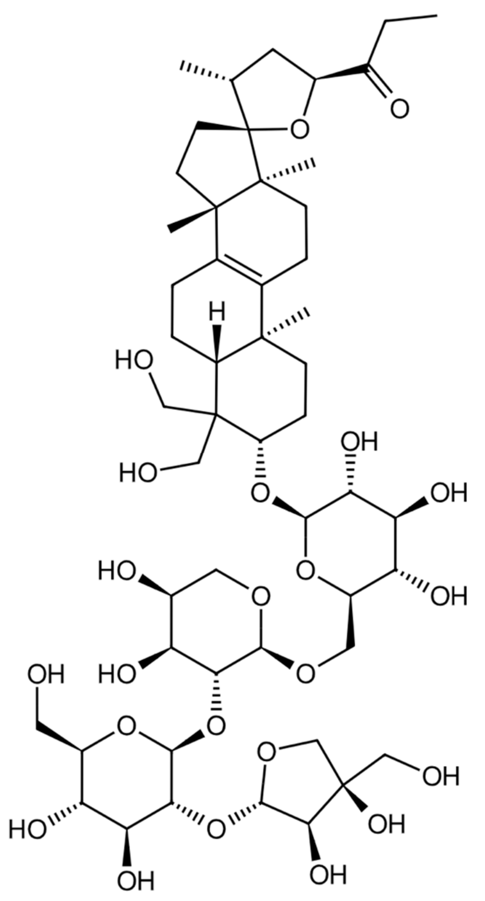

References

1. Zengin, G., et al., *Characterization of phytochemical components of Ferula halophila extracts using HPLC-MS/MS and their pharmacological potentials: A multi-functional insight.* Journal of Pharmaceutical and Biomedical Analysis, 2018. **160**: p. 374-382.
